# Computer assisted electromagnetic navigation improves accuracy in computed tomography guided interventions: A prospective randomized clinical trial

**DOI:** 10.1371/journal.pone.0173751

**Published:** 2017-03-15

**Authors:** Pierre Durand, Alexandre Moreau-Gaudry, Anne-Sophie Silvent, Julien Frandon, Emilie Chipon, Maud Médici, Ivan Bricault

**Affiliations:** 1 Department of Imaging, Radiology and Medical Imaging, University Hospital, Grenoble, France; 2 Laboratory of Techniques for biomedical engineering and complexity management – informatics, mathematics and applications, University Grenoble Alpes, Grenoble, France; 3 Laboratory of Techniques for biomedical engineering and complexity management – informatics, mathematics and applications, National Center for Scientific Research, Grenoble, France; 4 Clinical Investigation Center - Innovative Technology 1406, National Institute of Health and Medical Research, Grenoble, France; 5 Clinical Investigation Center - Innovative Technology 1406, Department of Public Health, University Hospital, Grenoble, France; 6 Clinical Investigation Center - Innovative Technology 1406, Research Department, University Hospital, Grenoble, France; Public Library of Science, FRANCE

## Abstract

**Purpose:**

To assess the accuracy and usability of an electromagnetic navigation system designed to assist Computed Tomography (CT) guided interventions.

**Materials and methods:**

120 patients requiring a percutaneous CT intervention (drainage, biopsy, tumor ablation, infiltration, sympathicolysis) were included in this prospective randomized trial. Nineteen radiologists participated. Conventional procedures (CT group) were compared with procedures assisted by a navigation system prototype using an electromagnetic localizer to track the position and orientation of a needle holder (NAV group). The navigation system displays the needle path in real-time on 2D reconstructed CT images extracted from the 3D CT volume. The regional ethics committee approved this study and all patients gave written informed consent. The main outcome was the distance between the planned trajectory and the achieved needle trajectory calculated from the initial needle placement.

**Results:**

120 patients were analyzable in intention-to-treat (NAV: 60; CT: 60). Accuracy improved when the navigation system was used: distance error (in millimeters: median[P25%; P75%]) with NAV = 4.1[2.7; 9.1], *vs*. with CT = 8.9[4.9; 15.1] (p<0.001). After the initial needle placement and first control CT, fewer subsequent CT acquisitions were necessary to reach the target using the navigation system: NAV = 2[2; 3]; CT = 3[2; 4] (p = 0.01).

**Conclusion:**

The tested system was usable in a standard clinical setting and provided significant improvement in accuracy; furthermore, with the help of navigation, targets could be reached with fewer CT control acquisitions.

## Introduction

Image-guided diagnostic and therapeutic procedures have become common practice for minimally invasive interventions. Ultrasound, X-ray fluoroscopy, Magnetic Resonance Imaging (MRI) and Computed Tomography (CT) are used to perform image guidance during interventions. Owing to fast volume acquisition, high resolution, good availability and low cost, CT guidance is a versatile approach for image-guided procedures.

The accuracy of needle placement is a critical step for the success of CT image-guided percutaneous interventions. Inaccurate needle placement can lead to loss of time, unwarranted X-ray exposure, adverse events, or failures such as non-diagnostic biopsy samples or incomplete treatment. Although CT guidance is very efficient for needle placement, some cases prove to be difficult; in particular, when an out-of-plane trajectory is required to achieve the anatomically safest route. The conventional workflow for freehand needle placement with CT guidance is detailed in [1]; this article also highlights how the result of the intervention can be highly dependent on its complexity and on individual physician skill.

In order to improve accuracy and limit X-ray exposure in CT-guided interventions, a range of CT navigation systems have been developed [1]. Some systems have been clinically tested by comparative [2–3] or randomized studies [4]. These navigation systems include a locator (optical or electromagnetic) which tracks in real-time the position of the needle.

In this paper, we present the results of a randomized clinical trial evaluating the IMACTIS^®^ CT electromagnetic navigation system prototype (Imactis SAS, La Tronche, France). This system allows the interventional radiologist to explore the patient’s anatomy in any plane, and visualize the planned needle trajectory before it’s insertion in real-time [5]. The results of a phantom study on out-of-plane punctures with this navigation system have shown a gain in time and accuracy compared to a standard CT-guided intervention [6].

The purpose of this clinical trial was to assess the accuracy and usability of this electromagnetic CT navigation system in a standard clinical setting, by comparing conventional and navigated procedures in a full range of routine CT interventions on the chest, abdomen, pelvis and bones, including biopsy, drainage, tumor ablation, sympathicolysis and joint infiltration.

## Materials and methods

### Trial design and participants

This open comparative randomized controlled prospective trial was approved by the ethics committee (Comité de Protection des Personnes, Sud-Est V, France) and was registered on ClinicalTrials.gov (NCT00828893—CTNAV).

The inclusion criteria were an indication for a percutaneous CT intervention without contraindications in adult patients with the ability to understand and sign the informed consent. Patients were included for whom the planned intervention was intended to be carried out using CT guidance exclusively, according to standard local practice and operator preference. Patients for whom the interventional radiologist had intended to use an additional imaging modality, such as ultrasound guidance, were not included in this study. Other exclusion criteria were patients with any implanted stimulation device, pregnant or breast-feeding women, unavailability of the navigation system or unavailability of a senior investigator.

**Fig 1:**
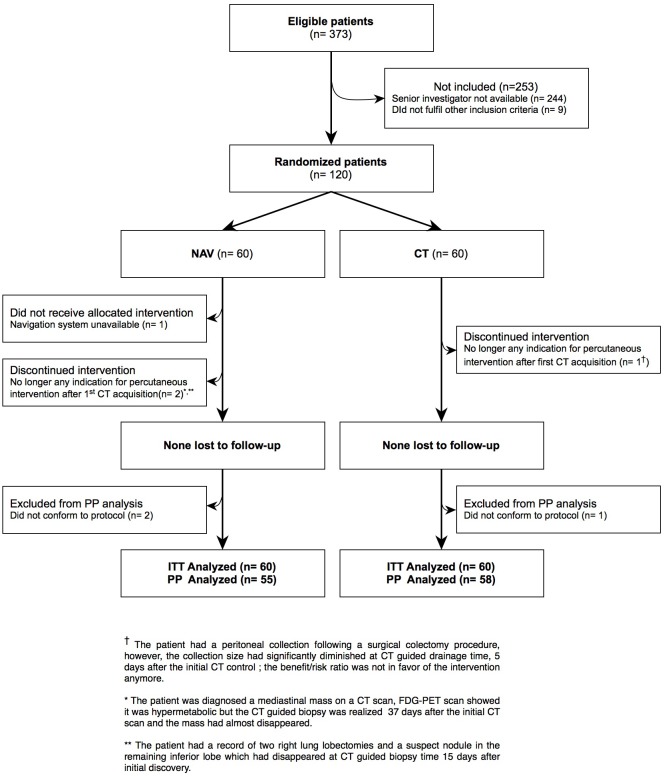
Patient flow.

From June 2010 to January 2012, 120 patients were included in the radiology and medical imaging department of Grenoble university hospital (Fig 1).

### Interventions

Operators were either senior interventional radiologists from our hospital with more than 5 years of practice, or resident under the direct supervision of a senior investigator. Operators were first trained to use the navigation system by carrying out two interventions on a phantom model.

All procedures were performed using a Brilliance 64 (Philips, Eindhoven, Netherlands) or Sensation 16 (Siemens, Erlangen, Germany) CT scanner. For the conventional CT intervention, the needle entry point was located using the CT gantry laser and a grid or a metallic wire placed on the patient’s skin.

For navigated interventions, we used the IMACTIS^®^ CT navigation system prototype. The IMACTIS system was composed of a station with a touch screen and a proprietary electromagnetic locator. This locator is composed of a magnetic receiver, located inside a needle holder, and a magnetic transmitter, designed to be fixed to the patient’s skin and detected in CT images, allowing an automatic registration between magnetic and CT coordinates. Once a CT-scan series (slice spacing of 2 mm maximum) had been transferred to the Navigation system, registration was performed without the need for any human interaction. Total DICOM series transfer and registration time was less than 20 seconds.

The position and orientation of the needle holder and the needle path were then displayed in real-time on two 2D reconstructed CT images extracted from the 3D CT volume. The two 2D images were computed such that the needle path was always displayed in the images; the system automatically chose the 2 perpendicular para-axial, sagittal or coronal oblique planes that optimize the visualization of the needle path. The needle holder was not tool specific and was suitable for any commercially available tool ranging from 25 to 11 gauge (needles, coaxial biopsy needles and guns, ablation devices, drainage kits). A video presenting the use of the navigation system can be viewed in [5].

### Outcomes

**Fig 2:**
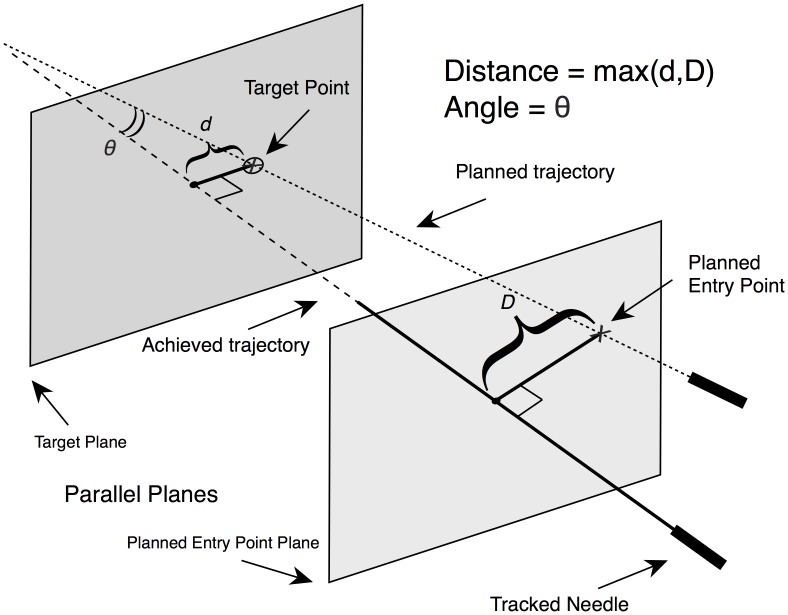
Main outcome computation. The accuracy of the initial needle placement is computed as the maximum distance between the planned trajectory and the achieved trajectory of the needle: Distance error = max(d,D), with d = distance between the achieved trajectory and the Planned Target Point, D = distance between the achieved trajectory and the Planned Entry Point. The achieved trajectory is defined as the straight line which passes through the actual position of the needle extracted from the first control CT acquisition, i.e. the straight line passing through the needle skin entry point and the needle tip (cf. Fig 3). This maximum distance error is computed along the entire real needle path, as if no iterative needle trajectory adjustments were made after the initial needle placement and as if the needle was pushed straight ahead up to the target depth. Thus it is related to the initial risk that neighboring critical tissue would be punctured by the needle, or that the target would not be reached by the needle tip. The angle θ between the planned trajectory and the needle is also computed.

**Fig 3:**
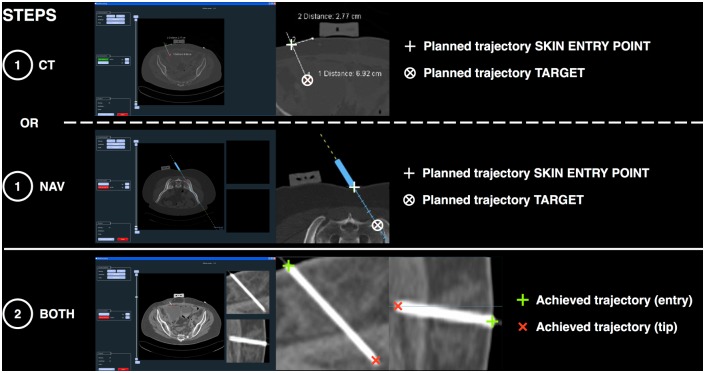
Examples of image data processing during main outcome computation. Distance and angle measurements are performed in two steps using a dedicated software: 1: The planned trajectory (i.e. planned entry point and planned target) is extracted from images saved by the operator before the needle is placed, showing the chosen trajectory that the operator will try to reproduce when placing the needle. The operator saves the planned trajectory either on the CT console (CT group) or on the navigation system (NAV group). 2: Once the needle has been positioned, the achieved trajectory is extracted from the first control CT acquisition showing the actual position of the needle. Multiplanar image reconstructions allow a fine selection of the points that define the entry point and the tip of the needle. The distance and angle between the planned and achieved trajectories (cf. Fig 2) are then computed using the software and saved for statistical analysis.

The main outcome was the accuracy of the initial needle placement, defined as the maximum distance and angle (Fig 2) between:

- the planned (expected) trajectory chosen by the operator (saved in the Navigation system log for the NAV group or saved in the CT scan console for the CT group), and
- the achieved needle trajectory, shown by the control acquisition performed immediately after the initial needle placement (Fig 3).

As part of this study protocol, the operator was instructed to position the needle with the highest accuracy possible from the first attempt, since it should reflect as accurately as possible the final planned trajectory.

**Fig 4:**
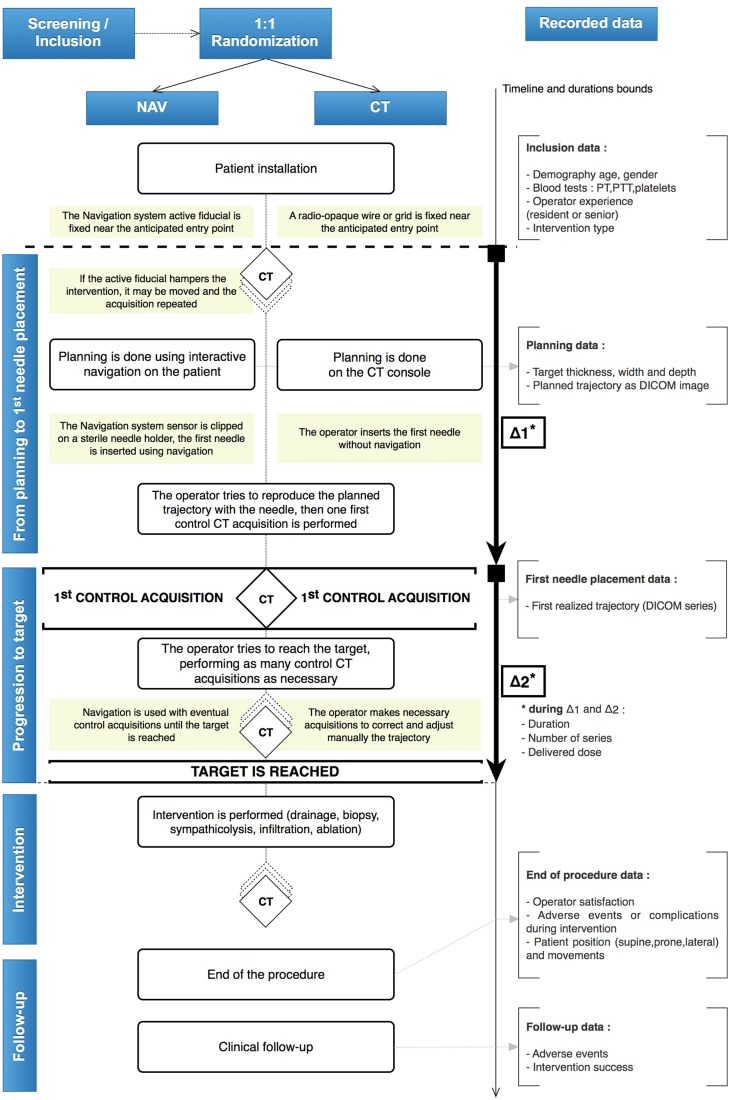
CTNAV trial protocol. The operator tries to reproduce as accurately as possible the planned trajectory using a navigated procedure (NAV group) or the standard procedure (CT group). The main outcome is the difference in distance and angle between the achieved and the planned trajectories.

Secondary endpoints were:

- The duration of the procedure, the number of control acquisitions performed and the delivered X-ray dose during Step Δ_1_ (from planning to initial needle placement) and during Step Δ_2_ (progression to target) (Fig 4).
- Operator satisfaction, reported by the operator after the procedure and before calculation of the main outcome, according to subjective criteria (overall satisfaction, accuracy appreciation, perceived ability to achieve an out-of-plan trajectory, and confidence).
- The short-term success of the procedure, reported by the operator by assessing if the planned target had been reached or not.
- Adverse events, reported using the Society of Interventional Radiology (SIR) scale [7–8].

During CT-guided interventions, the final distance between the needle tip and the target is the result of an iterative process, as the operator will frequently:

- evaluate the position of the needle relative to the target using a CT control image
- adjust the needle position and orientation
- move the needle forward (or even backwards if the trajectory is excessively incorrect).

In this study, we evaluated this global targeting process using two main indicators:

- The main outcome computes how precisely the operator has positioned his needle with respect to the planned trajectory, calculated from the initial needle placement.
- A secondary outcome records the number of iterations required to finally reach the target.

Indeed, one can expect that if the initial needle placement is precise, the number of iterations will be reduced: thus both criteria are expected to behave similarly.

These accuracy criteria are consistent with those chosen for previously published studies evaluating navigation devices in interventional radiology (in particular, cf. Figure 4 of reference [9]).

### Sample size

During a preclinical assessment of the navigation system described in [10], 72 “one-shot” oblique punctures were performed on a phantom model, half with and half without the use of the navigation system. The observed primary outcome was the error distance between the planned trajectory and the trajectory achieved by the needle. This preclinical study showed an average error distance of 6 mm with and 13 mm without the use of the navigation system, with standard deviations of respectively 4 and 11.3 mm.

The number of patients to be included in the clinical study was calculated using the preclinical study results. The following assumptions: μCT = 13 (mean distance observed in the CT group), μNav = 6 (mean distance observed in the NAV group), σ = Max(11.3; 4) = 11.3 (standard deviation chosen as the maximum standard deviation observed in the two groups, in order to maximize the number of patients included in the study), alpha risk α fixed at 0.05, statistical power fixed at 0.9, determined 56 as the number of patients to be included per group, i.e. a theoretical total of 112 patients to include. Assuming the number of inclusions lost to be 5%, the number of patients to be included was determined to be 59 per group, approximately 120 in total.

### Randomization and procedure

A senior investigator assessed patient eligibility, informed the patient about the study and obtained his written consent for inclusion. A senior investigator and the operator (if not the same person) discussed and rated by consensus agreement the expected technical difficulty of the intervention as “easy” or “difficult” based on the referral letter and review of previous imaging records available, in order to establish the proximity of sensitive anatomical structures such as vessels, at-risk non-target organs, etc.

Randomization was performed using a website, developed by a specialist society (FDA 21 CFR part 11). This platform was available 24h/24 for investigators. We considered the expected technical difficulty of the intervention to be a prognostic factor of the success of the gesture. The randomization was therefore stratified according to the two levels of predicted difficulty, to ensure comparability for the subgroup statistical analysis. Computer-generated stratified randomization lists were generated using a block factor of two (6%) and four (94%). These random block sizes were chosen to be small, in order to prevent group imbalance in the case of an early interruption of the study. The investigators were evidently blinded to the block size selected for the randomization process.

### Statistical methods

Continuous variables were expressed as medians [P25%, P75%] and compared using a Mann & Whitney non-parametric test. Categorical variables were compared using the Chi-square test or the Fisher exact test when necessary. Data were analyzed according to both the worst case scenario intention-to-treat (ITT) and per protocol (PP) principles. The robustness of significant factors from univariate analysis was confirmed by ANOVA analysis taking into account predefined confounding factors: stratification (easy/difficult intervention), operator experience (resident/senior), number of interventions made by the operator (frequent/occasional, if more/less than 15 interventions were carried out by the radiologist during the study), type of intervention (biopsy, drainage, etc.), patient’s position on the CT table, and presence of patient movement reported during the procedure. A Bartlett’s test, Levene’s test or data log transformation was carried out to confirm the data homoscedasticity for multivariate analysis. All p values were two sided. For the main outcome, multiple comparisons were made (distance/angle, ITT/per protocol, uni/multivariate). Following the Bonferroni correction method to counteract the problem of multiple comparisons, the statistical significance level was set at 0.00625 (0.05/8). For the secondary endpoints, it was set at 0.05.

Three subgroup analyses were performed according to the type of intervention (easy/difficult), the experience of the operator (senior/resident) and the number of interventions made by the operator (frequent /occasional). Analyses was performed using GNU R software (2.15.0).

## Results

### Patient flow

We enrolled 120 patients between December 2009 and January 2012 (Fig 1). The protocol and defined outcomes were not modified during the study, which proceeded as designed with the planned number of inclusions.

During the inclusion period, a total of 373 percutaneous CT interventions were performed at our center, thus, 32% of the interventions were included based on compliance with inclusion and exclusion criteria. Seven patients (NAV: 5; CT: 2) were excluded from the per-protocol (PP) analysis; no adverse event was reported for any of these seven patients: 3 for whom the initially indicated gesture was no longer indicated and was cancelled, one for whom the navigation equipment was unavailable and 3 for whom the protocol-required data collection was not complete.

These 7 patients were however included in the intention-to-treat (ITT) analysis; missing data was completed in the most pessimistic way for the NAV group and in the most optimistic way for the CT group.

### Baseline data

Table 1 gives the baseline characteristics of the 120 patients included in the ITT analysis, distributed according to the randomization.

**Table 1:** Baseline characteristics of the patient population.

		NAV group (n = 60)	CT group (n = 60)
Factors			
Demographics			
	Age^†^	59±16.3	62.2±13.5
	Sex-ratio (M/F)	33/27	38/22
Blood tests (NA = 22)			
	Platelets (G/l)*	327 [232; 415]	256 [210; 415]
	PT (%)*	86 [76.2; 95]	83.7 [71.8; 96.5]
	PTT (s, ref = 32s)*	34 [31.2; 38]	35 [32; 42]
Stratification and Operator			
	Easy vs. Difficult	38 vs. 22	37 vs. 23
	Senior vs. Resident Operator	40 vs. 20	35 vs. 25
	Occasional vs. Frequent Operator (≤15 / >15 interventions)	26 vs. 34	29 vs. 31
Intervention type (NA = 3)			
	Drainage	21	24
	Biopsy	21	17
	Sympathicolysis	7	7
	Infiltration	4	6
	Tumor ablation	5	5
Target characteristics			
	Thickness (mm)*	27.5 [10.8; 49.2]	28.5 [12; 45.5]
	Width (mm)*	27.5 [12.8; 52]	24 [13; 45.5]
	Depth (mm)*	53.6 [38.6; 81.3]	66.4 [51.8; 89.2]

^†^ Mean±standard deviation

*Median [P25% P75%]

NA: Not Available

Nineteen different operators (11 seniors, 8 residents) participated in the study.

The median number of interventions per operator was 3 [1, 7]. Among the total number of 60 interventions in each group, the proportion of interventions performed by “frequent” operators (those that carried out more than 15 interventions during the study) was comparable in both groups (NAV: 34/60; CT: 31/60 p = 0.71).

The proportion of interventions performed by senior operators was comparable in both groups (NAV: 40/60; CT: 35/60 p = 0.45). The proportion of interventions performed by senior operators was also comparable in the subgroups according to the expected difficulty of the gesture (“easy”: 44/75 “difficult”: 31/45 p = 0.33). Interventions included both scheduled and emergency procedures, and covered drainages, biopsies, tumor ablations (radiofrequency, microwave and cryotherapy), sympathicolyses and infiltrations.

No technical dysfunction of the navigation system was reported.

### Main outcome

With a median distance and angle error between planned and achieved trajectories of 4.1 mm [2.7; 9.1] and 4.7° [2.4; 8.2] in the NAV group *vs*. 8.9 mm [4.9; 15.1] and 7.9° [5.9; 13.2] in the CT group (p<0.00625), both ITT and PP analyses (Table 2) demonstrated a significant improvement in accuracy when the navigation system was used. ANOVA analysis demonstrated that these results were persistent after adjustment according to the identified confounding factors (Table 3).

**Table 2:** Main outcome ITT^†^ and PP results.

		NAV group	CT group	p-values (univariate analysis)	p-values (multivariate analysis)
***Intention to treat***					
	Analyzed population^†^	n = 60	n = 60		
	Distance error (mm) *	4.1 [2.7; 9.1]	8.9 [4.9; 15.1]	<0.001	<0.001
	Angle error (°) *	4.7 [2.4; 8.2]	7.9 [5.9; 13.2]	<0.001	<0.001
***Per protocol***					
	Analyzed population	n = 55	n = 58		
	Distance error (mm) *	3.8 [2.7; 8.3]	9.5 [4.9; 15.2]	<0.001	<0.001
	Angle error (°) *	4.1 [2.2; 7.6]	8.2 [6.1; 13.3]	<0.001	<0.001

^†^Patients whose data were unavailable were analysed using the worst case scenario: using the most pessimistic available data for the patients in the NAV group (n = 5) and the most optimistic available data for the patients in the CT group (n = 2)

* Median [P25%-P75%]

**Table 3:** ANOVA results for the main outcome (ITT).

Confounding factors	Distance (mm) (p values)	Angle (°) (p values)
Group (NAV/CT)	<0.001	<0.001
Difficulty of gesture (Easy/Difficult)	0.22	0.58
Senior vs. Resident Operator	0.06	0.04
Occasional/Frequent Operator (≤15 / >15 interventions)	0.07	0.17
Intervention type^†^	0.21	0.72
Patient position*	0.14	0.62
Reported patient movement during intervention (Yes/No)	0.5	0.7

^†^ Drainage/biopsy/others

* Supine/prone/lateral

**Fig 5:**
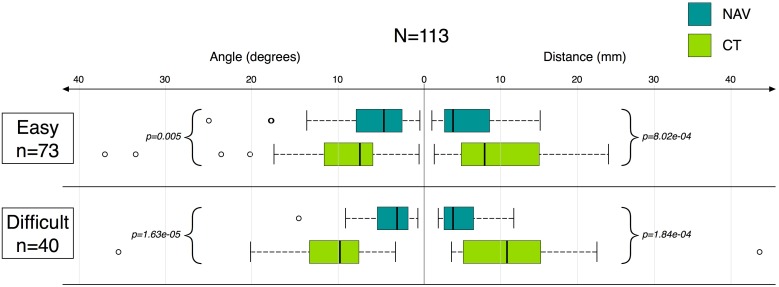
Angle and distance between the planned and the achieved trajectories according to NAV/CT group and intervention difficulty.

The gain in accuracy in the NAV group remains significant in both the easy and difficult intervention subgroups (Fig 5). A subgroup analysis based on operator experience shows an improvement in accuracy (with different ranges) when using the navigation system for both resident and senior operators. For residents, the median distance error was 5.5 mm [3.2; 10.1] in the NAV group *vs*. 10.4 mm [4.9; 17.7] in the CT group (p = 0.01). For seniors, the median distance error was 3.6 mm [2.6; 7.2] in the NAV group *vs*. 8.3 mm [4.9; 13.1] in the CT group (p<0.0001). The same conclusion was made for the subgroup analysis according to the number of interventions, showing an improvement in accuracy when using the navigation system for both frequent and occasional operators. In the subgroup of occasional interventions (≤15), the median distance error was 3.4 mm [2.7; 8.3] in the NAV group *vs*. 5.1 mm [4.2; 12.8] in the CT group (p = 0.02). In the subgroup of frequent interventions (>15), the median distance error was 3.9 mm [2.9; 7.9] in the NAV group *vs*. 11.8 mm [7.6; 16.8] in the CT group (p<0.0001).

### Secondary endpoints

#### Procedure duration, number of CT acquisitions and radiation dose

The duration of step Δ_1_ (from planning to initial needle placement) was lower in the CT group than in the NAV group (p = 0.001). No significant difference in the delivered X-Ray dose, or in the number of CT acquisitions during step Δ_1_, was demonstrated between the groups (Table 4).

**Table 4:** Procedure duration, number of CT acquisitions and radiation dose* (PP).

		NAV group (n = 55)	CT group (n = 58)	p-values
***From planning to initial needle placement (Δ1)***				
	Procedure duration (min)	22 [17; 26]	16 [14; 20]	0.001
	Number of CT acquisitions	1 [1; 2]	1 [1; 1]	0.077
	Radiation dose (DLP mGy.cm)	487 [320; 682]	467 [367; 668]	0.746
***Progression to target (Δ2)***				
	Procedure duration (min)	8 [6; 12]	7 [6; 13]	0.797
	Number of control CT acquisitions	2 [2; 3]	3 [2; 4]	0.01
	Variance of the number of control	σ²_easy_ = 1	σ²_easy_ = 1.11	
	Variance of CT acquisitions	σ²_diff._ = 1.1	σ²_diff._ = 6.36	
	Variance inhomogeneity p-value (easy *vs*. difficult interventions)	p = 0.59	p = 0.02	
	Radiation dose (DLP mGy.cm)	225 [133; 377]	297 [173; 449]	0.076

*Median [P25%-P75%]

Concerning the progression to target (step Δ_2_) (Table 4), no significant difference in the delivered X-Ray dose or in the duration was found between the two groups. However, significantly fewer control acquisitions were necessary to reach the target in the NAV group than in the CT group (p = 0.01). We performed an exploratory non-parametric variance homogeneity analysis for this outcome, according to the difficulty subgroup. The difference in the variance of the number of control acquisitions between easy and difficult interventions was significant in the CT group (p = 0.02) but not in the NAV group (p = 0.59), showing greater predictability in the number of control acquisitions needed for NAV interventions (in addition to fewer control acquisitions).

#### Satisfaction

Overall operator satisfaction was higher in the NAV group (Table 5) than in the CT group. The operators also considered their intervention to be more accurate, and perceived more freedom to perform out-of-plane trajectories in the NAV group. Confidence during interventions showed no significant difference between the two groups.

**Table 5:** Operator satisfaction after intervention (PP).

	NAV group (n = 55)	CT group (n = 58)	p-values
Overall satisfaction†*	9[8; 9.5]	8[7; 9]	0.025
Accuracy subjective appreciation†*	9[8; 9]	7[6.25; 8.75]	<0.001
Perceived ability to achieve out-of-plane trajectory (yes/no)	49/6	41/17	0.03
Confidence during intervention scored as:			0.16
• High	19	11	
• Normal	29	36	
• Low	7	11	

*Median [P25%-P75%].

^†^Satisfaction on a scale from 0 to 10.

#### Intervention success and adverse events

Among the total number of 117 interventions included in the per-protocol analysis (55 for NAV, 58 for CT), the reported immediate success of interventions was similar: NAV = 53/55, CT = 57/58 (p = 0.612). Three cases were registered as failures. Four adverse events were reported (NAV: 3/55; CT: 1/58). Failures and adverse events are detailed in Table 6.

**Table 6:** Failures and adverse events.

Type	Patient #	SIR grade	Age	Sex	Stratifi-cation	Group	Operator	Type of gesture	Comment
**Adverse event**	#3	B	54	F	Difficult	CT	Resident	Biopsy	The patient had a periadrenal hematoma after a biopsy, without complication or need for additional treatment.
**Failure**	#37	-	33	M	Difficult	CT	Senior	Biopsy	The patient had a 13 mm suspicious lung nodule, located behind a rib. The operator made two unsuccessful attempts to reach the target, and finally decided to stop the CT-guided intervention. The patient was subsequently referred for surgery.
**Failure**	#45	-	58	M	Difficult	NAV	Senior	RF Ablation	The patient was referred for RF ablation of a liver metastasis of a colon cancer. At the end of the intervention, the operator was retrospectively not completely sure that the ablated zone had been ideally located, he therefore preferred to register the result as a potential failure, and to plan a strict follow-up. Finally, no local recurrence was observed during the 25 month follow-up of this patient.
**Adverse event**	#51	B	24	M	Difficult	NAV	Senior	Drainage	24h after a peritoneal drainage, the patient reported abdominal pain and was successfully treated with analgesics.
**Failure Adverse event**	#84	C	69	M	Easy	NAV	Senior	Biopsy	The patient was referred for a lung nodule biopsy. He presented a pneumothorax during needle progression and the operator decided to interrupt the intervention before the biopsy could be performed. A puncture and aspiration with a chest tube were necessary.
**Adverse event**	#111	E	34	F	Difficult	NAV	Senior	RF Ablation	After hepatic tumor ablation using radiofrequency, the patient presented a pneumothorax, which required surveillance, and a severe right thigh skin injury beneath a misplaced radiofrequency ground pad.

A Fisher exact test was used to test the difference in frequency of adverse events between the two groups and showed no significant difference (p = 0.355).

**Fig 6:**
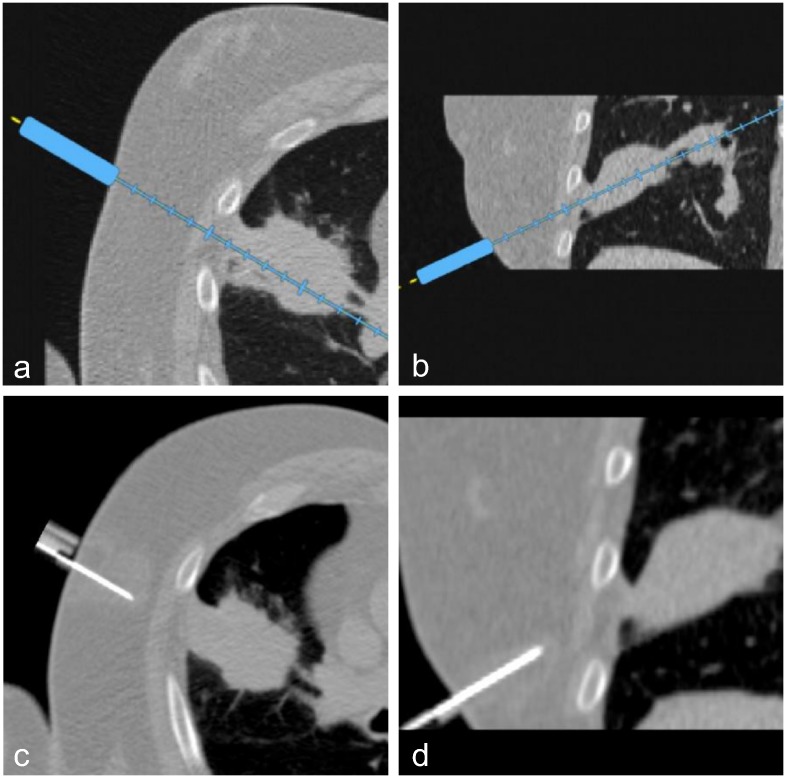
Illustration case (NAV). This 65 year old male patient had a record of lung carcinoma with recurrence after chemotherapy. There was a collegial indication for a percutaneous CT-guided biopsy for immunohistochemical (IHC) characterization. The patient gave written informed consent. The operator (senior) predicted an easy intervention. The patient was randomized to the NAV group. Planning was performed using the navigation system directly on the patient, with a double angulated intercostal trajectory (a, b). The first control acquisition showed the achieved trajectory (c, d) to be very similar to the planned trajectory (step Δ_1_). Two more control acquisitions were necessary to reach the target (step Δ_2_), and perform the biopsy. No adverse event was reported. Pathologic findings on the biopsy sample showed an Anaplastic Lymphoma Kinase (ALK) negative acinar adenocarcinoma of the lung.

**Fig 7:**
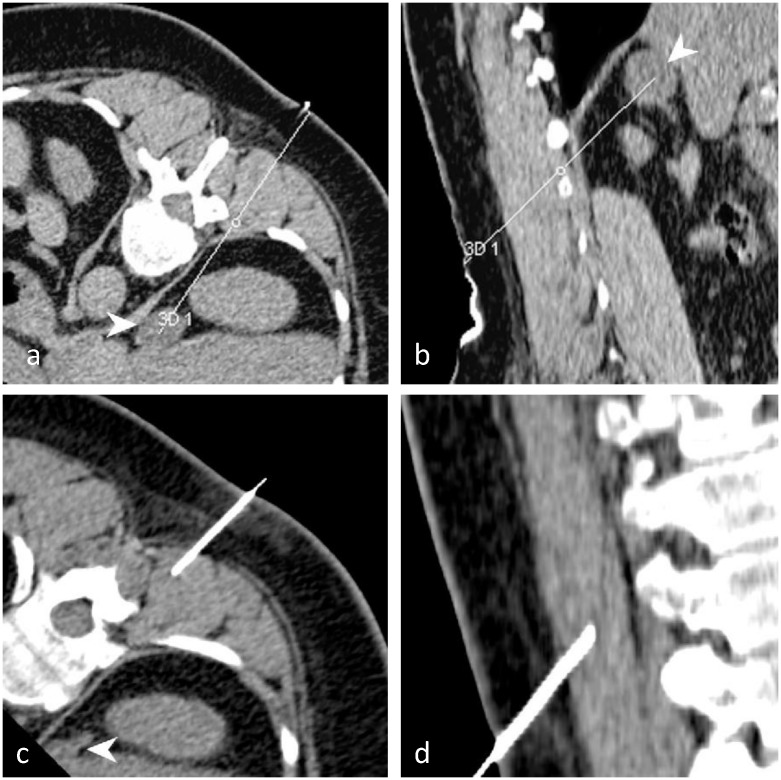
Illustration case (CT). This 54 year old female patient had a growing right adrenal gland mass (white arrow) and an indication for a percutaneous CT-guided biopsy. She gave written informed consent. The operator (resident) in agreement with the supervising senior operator predicted a difficult intervention, due to the necessity of a double angulated trajectory. The patient was randomized to the CT group. The planned trajectory was saved on the CT console. The control CT acquired after the initial needle placement (step Δ_1_) showed a large difference between the planned (a, b) and the achieved trajectories (multiplanar reconstructions) (c, d). Twelve more attempts and iterative CT control acquisitions were required to reach the target (step Δ_2_). After biopsy, the last control acquisition showed a small periadrenal hematoma without the need for additional treatment. Pathologic findings on the biopsy sample showed adrenal tissues with metastasis from a malignant lung tumor.

Two examples illustrate the results for the NAV and CT groups (Figs 6 and 7).

## Discussion

### Results interpretation

The present study demonstrates a gain in accuracy when using the IMACTIS^®^ CT navigation system prototype. The gain was consistent irrespective of whether the operator was a resident or a senior, whether the operator performed few (≤15) or many (>15) interventions, and whether the procedure was anticipated as being easy or difficult. In the CT group, the accuracy was significantly lower, and worsened when interventions were difficult (Fig 5). In interventions without navigation, as expected, the resident operator median error was higher than the senior operator error. However, when using the navigation system, residents obtained median errors comparable to those obtained by senior operators without navigation, while median errors for seniors were significantly lowered when using the navigation system.

The intervention preparation phase was longer (Table 4 –Δ_1_) in the NAV group compared to the CT group. This could be explained by:

- The time required to install the prototype. An optimization of the equipment would facilitate the use of the system in routine clinical practice, in particular the ergonomics of the needle holder, and an improved integration of the NAV system installation into the routine procedure workflow.
- The number of participating radiologists (19 seniors and residents). Some operators used the navigation system for only one intervention (median number of interventions = 3). This lack of experience lengthened the average preparation phase, however it was shown that even novices could achieve good performances using the navigation system.
- The navigation system enabled the radiologist to explore many possible trajectories. Standard trajectories were explored, as well as more complicated trajectories (with double angulation for example), resulting in longer average preparation phases. An interesting further study could determine if this improved planning capability resulted in more optimal, safer trajectories.

The use of the navigation system allowed the operators to reach the target with fewer control acquisitions (Table 4 –Δ_2_). Operators in the NAV group felt they had greater accuracy (Table 5) and thus progressed more quickly and more safely to the target, reducing the number of intermediary control acquisitions. Moreover, since navigated interventions were indeed more accurate, fewer corrections of erroneous trajectories were required, compared to conventional CT. When interventions were difficult, there was a significantly higher risk in the CT group that a large number of control acquisitions would be required. The variance inhomogeneity analysis in Table 4 supports this point.

In accordance with fewer control acquisitions in the NAV group, Table 4 shows that the X-ray dose delivered by the controls acquired during the progression to target was smaller using NAV (225 [133; 377] mGy.cm) than using CT (297 [173; 449] mGy.cm). However, this difference did not reach the level of statistical significance (p = 0.076). In our center, globally, few CT controls were made by the operators (mean value of 3 controls in the non-navigated group; vs. 10.3 controls for example in reference [3]), this could have rendered more subtle the irradiation difference between the CT and NAV groups. A multicentric study would therefore be interesting to further explore the positive impact of navigation on irradiation. The use of low dose protocols associated with navigation could even contribute to a greater reduction of X-ray delivery [11].

Even if the median time taken to reach the target was comparable between the NAV and CT groups (median <10 min), this time was more consistent and predictable in the NAV group where time to reach the target was always <30 min with a low standard deviation, while 2 interventions in the CT group required more than 45 min before the target could be reached (step Δ_2_). These outliers, encountered for difficult interventions in the CT group, are potential sources of discomfort and high X-ray exposure for the patient, stress for the operator and may lead to poor optimization of imaging resources.

All the reported adverse events were procedure related and are common complications (Table 6). None of these adverse events could be directly linked to the use of the navigation system, and the difference in frequency between NAV and CT interventions was not significant.

The system tested was reliable, no system dysfunction was reported, the automatic registration and detection of the magnetic transmitter attached to the skin was always successful and navigation using the needle holder was always possible.

### Comparison with other navigation systems for the assistance of CT-guided interventions

A randomized paired trial has shown that a laser guidance system can improve the accuracy, the number of control CT acquisitions and the time to place the needle [12]. Yet, such laser-based guidance systems do not have complete navigation capabilities, including real-time tracking of needle movements. Moreover, these systems impose that the patient remains perfectly immobile between trajectory planning and needle placement.

Various navigation systems for planning and real-time needle tracking have been developed and evaluated. These systems are based either on optical [3,13,14,15] or magnetic [4,16,17,18] localization; the specific advantages and disadvantages of both systems in different applications are compared in [13]. The main disadvantage of optical systems is that a direct line of sight between the cameras and the markers attached to the instrument is required, which can limit the ergonomics of such systems and their applicability in a radiological clinical setting.

Navigation systems using electromagnetic tracking do not suffer from line-of-sight constraints, but their performance can be affected by the presence of metal or other magnetic perturbations.

In [3], the authors performed a comparative, non-randomized study of an optical navigation system, showing that the number of control CT acquisitions, the amount of radiation and the duration of the procedure was significantly lower in the navigation group. However, it should be noted that each of the 3 experienced operators were required to perform 20 procedures before they could include patients in the study.

Authors of [5] reported a randomized controlled trial evaluating the use of an electromagnetic navigation system in addition to CT fluoroscopy in 60 consecutive patients undergoing a lung biopsy. Navigated procedure results were not statistically different to standard CT procedure results for fluoroscopy time, radiation dose, number of needle repositions, incidence of pneumothorax, need for chest tube, or diagnostic yield. Among the 26 patients randomized for navigation, 8 navigated procedures (31%) failed because of technical failure or incoherent localization data, suggesting that the particular electromagnetic navigation system tested in this study might not have been ready for routine clinical use.

Finally, navigation systems offering the possibility to fuse several imaging modalities have been developed [9, 19–22]. These systems can fuse pre-operative images (for instance, CT imaging) with real-time intra-operative images (such as ultrasound). The registration between pre-operative images and the navigation system is often based on interactively designated anatomical landmarks, or fiducials placed on the patient that are localized with a mouse click on pre-operative images and palpated magnetically; this step can be tedious and may lack ergonomics and reproducibility.

In [20], a navigation system capable of fusing US and CT images has been evaluated in the particular context of liver biopsies under exclusive CT guidance. This randomized 50 patient trial showed an improvement when using the navigation system concerning the radiation dose and operation time; no information was given concerning accuracy. This study involved a single experienced investigator who had practiced on 20 phantoms and had used the system for 6 months before including patients in the study.

As detailed above and in [1], there now exists a wide variety of developed systems; yet few have reached commercial availability, and none so far have been integrated into routine clinical practice. This may be explained by the associated cost, the complexity of using the system, the lack of reliability or insufficient added medical value.

The navigation system tested in this study presents some favorable factors for its integration into routine clinical practice:

- Needle holder tracking is less expensive than needle tip tracking.
- This navigation system has been developed specifically to assist CT-guided interventions, allowing a dedicated automatized workflow that limits the system usage complexity.
- This study demonstrates the accuracy and reliability of this navigation system in clinical conditions, even for occasional and non-expert operators.

However, only a larger multicentric study and future clinical routine observations will be able to predict the real potential of this navigation system. One such study has already been designed and started [NCT01896219].

### Limitations of this study

This prospective randomized clinical trial demonstrated an improvement in accuracy when using the tested navigation system. This system proved to be reliable when evaluated in a routine clinical setting, by operators having diverse experience and no extensive training on the system, and for a large variety of types of interventions. Nevertheless, there are limitations to this study:

- It was not a multicenter trial. However, the large number of operators allowed a broad evaluation of the system. As the navigation system was not evaluated by a small number of highly trained expert radiologists, we can expect that the results of this study are more easily transposable to a general population of non-specialized radiologists.
- Operator experience could have been used as a stratification variable in order to have a better guaranty regarding the comparability for this criterion. Nevertheless, owing to randomization, the distribution of senior operators proved to be comparable in both groups.
- Inclusions were neither systematic nor consecutive. Our cohort does not represent a random sample of our overall patient referral population. Inclusions mainly relied on the availability of a senior investigator.
- In order to avoid bias during this clinical study, the randomization was implemented after the inclusion of each patient, and clinicians were blinded to the details of the randomization process. However, it was not possible to conduct a double-blind study, as is often the case with clinical trials of medical devices. However, in both the CT and NAV groups, the main outcome was calculated automatically and identically, once the entry and end points of the planned and achieved needle trajectories had been registered in the study database (Fig 3). Furthermore, the validity of the registered coordinates of the needle tip and entry point, from which the main outcome is computed, has been checked by an independent observer, blinded to the CT or NAV group.
- The estimated duration of interventions may not necessarily represent those of routine interventions, because of use of the system by novice users, and because of data collection and the constraints imposed by the strict adherence to the trial protocol. Therefore, future trials might provide better estimations for procedure duration.
- The navigation system evaluated in this study was designed for the assistance of specific interventions performed exclusively with CT guidance, and it has no multimodality capability. Thus, this study cannot evaluate the interest of a fusion between CT and other imaging modalities, such as ultrasound.
- The navigation system evaluated in this study was designed to track a needle holder, and not to track the needle tip. Consequently, this system has no capability to compensate for needle bending during the intervention. This study demonstrates however that needle holder tracking provides a reliable localization and improves accuracy. Further studies comparing with needle tip tracking technology would be interesting.
- No respiratory motion tracking was provided for this navigation system; a breath-hold approach was therefore required for certain procedures. New technical developments would be useful to take into account possible respiratory motion, and possibly further increase the accuracy of the navigation system.

## Conclusion

In summary, though numerous navigation systems have been developed, few clinical studies have been conducted according to a robust methodology that are able to demonstrate an advantage in the use of these systems. Findings from this prospective randomized controlled trial suggest that the tested navigation system improves the accuracy of CT-guided interventions, particularly when they are difficult to perform. A specific interest of this trial is that the tested system has been evaluated in a routine clinical setting by operators having diverse experience and no extensive training on the system, and for a large variety of types of interventions. This is particularly valuable in the context of the increasing number of indications for CT-guided interventions.

## Supporting information

S1 File Clinical protocol in English. (PDF)

S2 File Clinical protocol in French. (PDF)

S3 File Consort 2010 checklist. (DOCX)
