# Supplementary material for: Computer assisted electromagnetic navigation improves accuracy in computed tomography guided interventions: A prospective randomized clinical trial
Source: PLoS One. 2017 Mar 15;12(3):e0173751. doi: 10.1371/journal.pone.0173751 (PMC5351986; doi:10.1371/journal.pone.0173751)
Supplement: S1 File — (PDF) [file pone.0173751.s001.pdf]

Pôle d'imagerie

EXPERIMENTAL PROTOCOL

1. GENERAL INFORMATION

**Title:** Interest of a navigation system to assist CT-guided interventions.<br>**Short Title:** CT-Nav		
**AFSSAPS Registration number: 2008-A00742-53**	**Sponsor's reference: 0812**	
**Date: 6 July 2010**	**Version: 11.4**	
**Study design:** Single center, prospective, open, randomized, controlled clinical trial with two parallel groups		
**Study Phase:** Equivalent to phase II/III of drug development		
**Medical Device to be studied:** Navigation system for CT-guided interventions.		
**Coordinator-Investigator authorized to sign the protocol:** Dr I. Bricault (Grenoble)<br>**Coordinator-Investigator:** Dr I. Bricault (Grenoble)		
**Investigators:**<br>Pr G. Ferretti<br>Dr C. Sengel	Dr JN. Ravey<br>Dr M. Rodière<br>Dr A. Jankovski	Dr L. Felix<br>Dr V. Cuvinciuc<br>Dr C. Dubois<br>Dr O. Eker
**Participants:** Interns in the University Radiology and Medical Imaging Clinic of Grenoble University Hospital		
**Other participants:** Dr A. Moreau-Gaudry (Clinical Investigation Center - Innovative Technologies, CIC-IT), Mr. Lionel Carrat (Imactis company), Ms. K. Guerrero (CIC-IT), Ms. AS Silvent (CIC-IT)		
**Study center:** Grenoble University Hospital – Imaging Pole – University Radiology and Medical Imaging Clinic (CURIM)		
**Sponsor:** Grenoble University Hospital (C.H.U. de Grenoble)<br>**Sponsor's representative authorized to sign the protocol**: Frédérick Marie<br>Direction de la Recherche Clinique et à l'Innovation, CHU de Grenoble, 38043 Grenoble Cedex 09<br>Tel: 04 76 76 59 57 Fax: 04 76 76 52 21		
**Study Coordinating Center:**<br>Clinical Investigation Center - Innovative Technologies – Inserm 803<br>CHU de Grenoble, 38043 Grenoble Cedex 09<br>Tel: 04 76 76 92 60 Fax: 04 76 76 92 62		
**Ethics committee approval (CPP Sud-Est V): 1 July 2009**		

CONTENTS

1. GENERAL INFORMATION ... 1
2. ABREVIATIONSAND DEFINITIONS ... 4
   2.1. ABBREVIATIONS ... 4
3. STUDY RATIONALE ... 4
   3.1. GENERAL PRESENTATION: ... 4
   3.2. GENERAL DESCRIPTION OF THE MEDICAL DEVICE ... 5
   3.3. RESULTS OF PREVIOUS STUDIES THAT ARE PERTINANT TO THIS BIOMEDICAL RESEARCH ... 7
4. OBJECTIVE(S) OF THE STUDY ... 7
   4.1. PRESENTATION AND OVERALL OBJECTIVE ... 7
   4.2. PRIMARY OBJECTIVE AND PRIMARY ENDPOINT ... 10
   4.3. SECONDARY OBJECTIVES AND SECONDARY ENDPOINTS ... 12
5. RESEARCH STUDY DESIGN ... 15
   5.1. STUDY TYPE ... 15
   5.2. GENERAL ORGANIZATION OF THE STUDY AND FLOW DIAGRAM ... 17
   5.3. STUDY SITE ... 19
6. CHARACTERISTICS OF SUBJECTS ... 19
   6.1. SUBJECT RECRUTEMENT ... 19
   6.2. INCLUSION CRITERIA ... 19
   6.3. NON-INCLUSION CRITERIA ... 19
   6.4. CONCOMITANT TREATMENTS ... 19
7. VARIABLES TO BE MEASURED AND METHODS ... 20
   7.1. CLINICAL PARAMETERS ... 20
   7.2. PARA-CLINICAL PARAMETERS ... 20
   7.3. DATA COLLECTED DIRECTLY IN THE CASE REPORT FORM ... 20
8. TREATMENT OR PROCEDURES TO BE COMPARED ... 20
   8.1. TEST TREATMENT ... 20
   8.2. REFERENCE TREATMENT ... 20
   8.3. RANDOM ALLOCATION METHOD (CENTRALIZED RANDOMIZATION) ... 21
9. DATA COLLECTION AND MANAGEMENT ... 21
   9.1. DATA COLLECTION ... 21
   9.2. MONITORING ... 21
10. STATISTICAL ANALYSIS ... 21

10.1. STEERING COMMITTEE 21
10.2. DATA ANALYSIS STRATEGY 21
10.3. CALCULATION OF NUMBER DE SUBJETS 22
10.4. RESPONSABILITY FOR ANALYSES 23
10.5. SITE OF DATA ANALYSES AND SOFTWARE 23

REFERENCES 24

2. ABREVIATIONSAND DEFINITIONS

2.1. Abbreviations

**CRA**	:	**Clinical Research Assistant**
**CHU**	:	**University Hospital**
**CIC-IT**	:	**C**linical Investigation Center – **I**nnovative Technologies
**CPP**	:	**C**omité de **P**rotection des **P**ersonnes = Ethics Committee
**CURIM**	:	**U**niversity **R**adiology and Medical Imaging Clinic
**INR**	:	**I**nternational **N**ormalized **R**atio
**APTT**	:	**A**ctivated Partial thromboplastin time
**CT**	:	**C**omputed Tomography
**PR**	:	**P**rothrombin Ratio

3. STUDY RATIONALE

3.1. General Presentation:

3.1.1. Problem posed

Initially used in radiographic mode and subsequently in CT, X-rays are invaluable for guiding the gestures made during percutaneous interventional radiology. While simple ultrasound guidance can be used for certain acts, many inaccessible or poorly echogenic lesions require the use of CT guidance. The purpose of these acts is either diagnostic (sampling for histological or bacteriological analysis) or therapeutic (foraminal infiltrations, drainage of pleural abscesses, treatment of tumors by radiofrequency, etc.).

The common denominator of all these procedures is the preceding step of local anesthesia, which consists of guiding a local anesthetic needle to a subcutaneous target.

The CT scan provides a set of images in the transverse plane: it is then easy to plan a trajectory in the plane of the transverse images. The operation becomes complicated when it comes to planning a trajectory that is oblique with respect to this plane due to the presence of vital structures (arteries, organs at risk) or obstacles (bone), as it requires analysis of multi-planar reconstructions from CT slices. Furthermore, the effective realization of this act is made difficult by the need to estimate complex angulation and visualize the path of the needle using a reconstruction and a "mental" projection of the anatomy of the patient. In some cases, due to this complexity, the operator is forced to choose a trajectory in the transverse plane, rather than an oblique trajectory, even though it is not optimal. Moreover, the more difficult it is to perform the planned act, the more intermediate CT controls are necessary, with a corresponding increase in the total radiation dose to the patient.

Using the scanner data, the navigation platform provides a 3D vision of the patient's anatomy and superimposes on it, in **real time**, the path of the puncture needle. The puncture needle is equipped with a magnetic receiver that can be located in 3D using a magnetic transmitter. The operator can thus visualize directly on a screen the path of the needle, both during the planning phase (performed simply by moving

the needle above the patient without penetrating the skin, thereby seeking an optimal path) as well as during the actual puncture. The operator sees a representation of the trajectory in three planes that are permanently aligned with the position of the needle, and possibly oblique. He/she thus avoids the constraints of conventional procedures, which pushes him/her to stay in the transverse plane of acquisition. In addition, the operator has control of his/her movements in real time without involving radiation.

In view of the constraints outlined above, the use of the navigation platform is expected to:

1- Increase trajectory possibilities
2- Increase the precision of the procedure
3- Decrease the duration of the procedure
4- Decrease the discomfort experienced by the patient
5. Decrease the total dose of radiation

The aim of this study is to evaluate the contribution of a recently developed navigation system to the previously identified problems of needle guidance.

3.1.2. Data in the literature

To our knowledge, there are no published data from clinical studies concerning the guidance of a needle during an interventional radiology procedure combining CT and a navigation system.
Navigation systems are already used in orthopedic and urological surgery. They are a great help for the surgeon enabling him/her to advance a needle or trocar in the patient's body with precise visualization of the surrounding anatomical structures. Computerized devices to aid biopsy have also been assessed with other imaging techniques such as ultrasound and fluoroscopy, particularly for percutaneous renal puncture.
The attractive alternative in interventional CT radiology is the use of fluoro-CT. This tool allows one to monitor the progress of the needle by performing X-rays. The radiologist can either perform continuous monitoring of the progress of the needle (dynamic mode), or make quick spot checks after each movement of the needle. In both situations, there is additional irradiation for both the patient and the radiologist.

3.2. General Description of the Medical Device

3.2.1. Identification

The medical device, not yet CE labeled, shown in paragraph 3.1.1, to be used in this biomedical research is composed of:

1- The navigation system
2- The specific navigation software for percutaneous puncture under CT control.
3- Navigation accessories:
   a. Sterilizable plastic needle holder;
   b. Plastic hybrid localizer equipped with a magnetic transmitter;
   c. Single use sterile covers to encapsulate magnetic transmitters and receivers;
   d. A USB key provided for each intervention in order to run the software and temporarily store the data necessary for the Biomedical Research.

3.2.2. Manufacturer

This device is manufactured by IMACTIS, located at 5, avenue du grand Sablon, 38700 LA TRONCHE, France; SIRET number: 509 967 030 00016, telephone: 0660502524 email: info@imactis.com ; website: http://www.imactis.com.

3.2.3. Installation and instructions for use

The navigation system is composed of a trolley equipped with a PC, a touch screen and a magnetic tracking system. It is placed in the radiology room during the procedure, as shown below. To use the navigation platform the following instruments are provided by IMACTIS®

- Needle Holder, sterilizable
- Hybrid localizer, non-sterilizable. This device can be inserted into a disposable sterile cover conventionally used for any non-sterilizable instrumentation

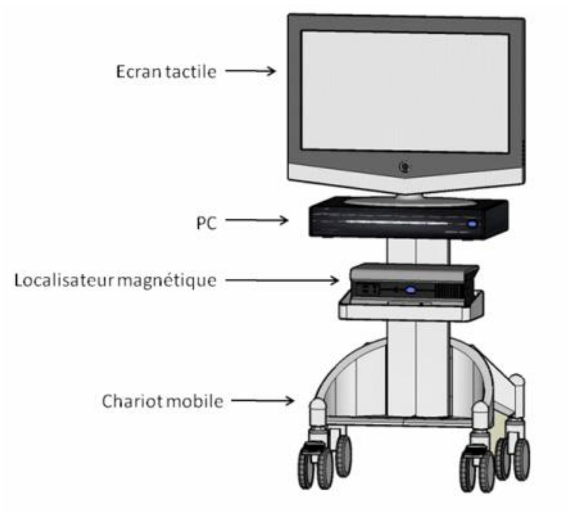	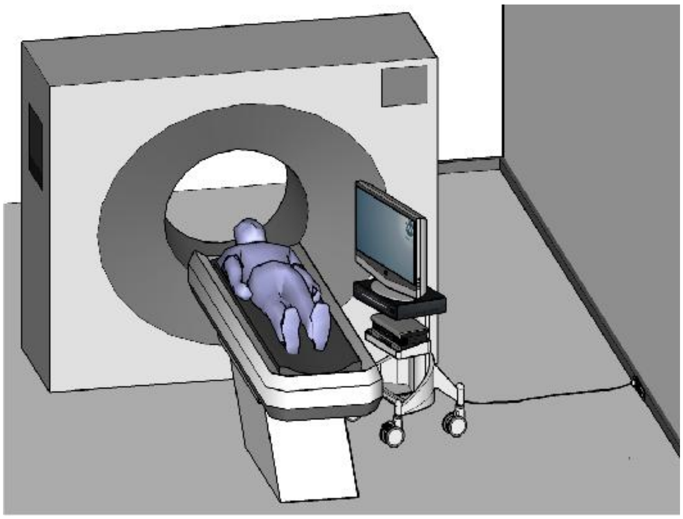
Navigation system	Navigation system in the interventional radiology room

The operating principle is based on the localization of the position of the needle relative to the patient in space and in real time, using a magnetic locator. Through the image registration, the scan of the patient can be replaced by the patient's actual anatomy. In short, the radiologist can visualize in real time the position of his instrument in the patient's CT images, as shown in the image below.

This principle is already used in clinical routine by orthopedists, ENT specialists and neurosurgeons

3.3. Results of previous studies that are pertinent to this biomedical research

A preliminary assessment of the system, with an optical tracking system, was made on a phantom in the interventional radiology room. These tests were performed on a phantom consisting of foam glued to a wooden board, pierced with holes of various diameters and inclinations. The radiologist had to attempt to traverse the foam and attain the holes under CT conditions. Two types of puncture were made: first, in the conventional way without using the navigation system, secondly, using the navigation. 36 punctures were performed in each group with different operators. The observed primary endpoint was the distance from the point of the needle to the target. This distance was measured by CT image control. The secondary endpoints explored were related to intervention times for different modalities (navigated/not navigated), and the angulation relative to the axial and sagittal planes.

This objective of this first step was to:
- Check in ideal conditions (on an inanimate object) the accuracy of the images provided by the navigation system by comparing them with those of the CT scan
- To become familiar with the system;
- To compare the accuracy of each method.

Early results have shown significantly better accuracy in terms of distance to the target (see Appendix A), angulation in the transverse axial plane and a gain in time with the use of the system.

A preliminary assessment of the system with the magnetic locator will also be conducted under the same conditions to confirm that the magnetic system is equivalent to the optical system and provides the same benefits. This assessment will naturally be attached to the technical file and will be addressed to the competent authorities.

4. OBJECTIVE(S) OF THE STUDY

4.1. Presentation and overall objective

According to current routine practice, all candidate patients for the study will have had imaging (ultrasound, MRI or CT) that revealed a lesion requiring an interventional, diagnostic or therapeutic procedure, under CT guidance. The procedure indication is a result of a discussion between radiologists, clinicians and surgeons and will proceed as follows:

• **The position** (supine, prone or side) of the patient on the table is determined by the radiologist according to the first imaging assessment. It essentially depends on the location of the target. It will take into account the patient's tolerance to this position.

• **The tracking step** (step 1) begins with the first CT acquisition and ends after the positioning of the local anesthetic needle. **In this study, the operator will be informed of the need for precision in the trajectory of the local anesthesia needle during the tracking phase since the latter will define by extrapolation the desired final trajectory.**

• **The intervention** (step 2) is carried out after the local anesthetic and consists of reaching with a needle, the target lesion or organ that is the subject of the intervention.

This study aims to compare the puncture trajectory performed with either the conventional method or with the navigation system. Each patient will randomly receive, either one or the other method, from the beginning to the end of the procedure. **We consider that only the tracking step is essentially identical between the two methods, and this, regardless of the patient or the interventional procedure performed**. For this reason, we define the primary endpoint as only the tracking step.

4.1.1. The conventional procedure (CT Group)

Before CT acquisition, a plastic object (identical to that used in the navigation process: the hybrid localizer) is placed on the patient. It allows a landmark to be defined from which measurements are taken to prepare for the intervention. On the CT scanner console the operator defines the optimal path she/he would like to obtain: it is this planning that will serve as a reference (***planned trajectory***). This trajectory is not necessarily in the plane of acquisition and might require reconstructions to be built. The image(s) to be used as reference will be **annotated** with the entry point and the target point. This(ese) image(s) will be **recorded.**

The slice that includes the trajectory and the skin entry point is noted on the scanner console. The manipulator marks the entry point with a felt-pen on the skin with the help of the CT tracking indicating the slice and measuring the distance between the metal object and the entry point. The radiologist, without visual aid, positions the local anesthesia needle after determining the precise point of entry and angle of penetration of the needle. Once the needle is in place, the first CT control scan is performed. The position of the local anesthesia needle in the CT image shows the ***actual trajectory*** (entry point and target point). The **planned and the actual trajectories** will then be compared.

Once the tracking step is complete, the intervention continues with puncture of the skin using the same technique used for the positioning of the local anesthesia needle. Once the puncture has been performed, a control CT scan is performed. The final position of the puncture needle in the CT image materializes the actual trajectory

4.1.2. The new procedure (NAV Group)

The steps of the procedure are as follows:

A - Preparation of the navigation system

1 / The navigation station is switched on and starts automatically

2 / The navigation software is started via the USB key

3 / The software checks the connection to the CHU computer network and the connection of the magnetic components

4 / Once the above checks have been made, the software waits to receive a CT scan.

B - Preparation of the instruments:

1 / The hybrid localizer fitted with a magnetic transmitter is removed from its packaging, ready to be attached in the vicinity of the area to be punctured.

2 / A magnetic sensor is attached to the non-sterile needle holder

3 / A magnetic sensor is inserted in the sterile drape and fixed onto the sterile needle holder.

C - Installation of the patient:

1 / Usual CT protocol
2 / At the end of installation, the hybrid localizer fitted with a magnetic transmitter is attached to the patient's skin near the area to be punctured.

D - Diagnostic scan:
1 / Usual protocol
2 / At the end of the CT acquisition, the scan is "transferred " onto the navigation system (ready to receive) by the computer network of the hospital following the standardized DICOM protocol.

E - Planning of surgical procedure
1 / The navigation software retrieves the CT scan and automatically performs the registration, a preliminary step necessary for the navigation phase.
2 / The radiologist uses the non-sterile needle holder to a) navigate in the patient's CT images, b) plan his/her procedure and c) identify the entry point on the patient's skin around which the sterile field necessary for the intervention will be placed.

F – Setting up the sterile field:
1 / Carried out according to the usual protocol.
2 / In addition, so as to work in optimal localization conditions, the radiologist positions the above hybrid localizer, in the work area, after having placed it in a sterile bag.
3 / A new CT scan focused on the work area is then performed. This examination is the reference for planning the procedure using the navigation system. Following this step, the patient and the radiologist are under sterile conditions.

G - Navigation step:
1 / The radiologist uses the sterile needle holder fitted with the magnetic sensor inserted in its sterile cover.
2 / The radiologist navigates in the patient's CT images under sterile conditions.

At any time, the radiologist may decide to perform a control CT scan to verify the correct positioning of his/her instrument. In this case, the loop described below will be carried out as many times as the radiologist wishes until the correct final positioning of the instrument is achieved:

H - Loop:
1 / Realization of a control CT scan
2 / The scan is transferred to the navigation system
3 / The navigation software retrieves the CT scan and automatically performs the registration
4 / The radiologist uses the sterile needle holder fitted with a magnetic sensor inserted in its sterile cover to navigate in the CT images.

In the case of a device failure, the radiologist may at any time abandon the navigated procedure and switch to the conventional procedure, without any consequences on the quality of his/her intervention and patient safety.

When performing this operating procedure, two alternatives are possible in step F when positioning the hybrid localizer necessary for the realization of the intervention in sterile conditions.

• **Variant 1:** Instead of moving the first hybrid localizer, the radiologist places a second localizer (for which sterility precautions have been taken) in the work area.

• **Variant 2:** the hybrid localizer from step C2 is immediately positioned for the realization of the intervention in sterile conditions (so as not to hinder the creation of the sterile field, and sufficiently close to the point to be punctured). It may be unnecessary to move and put it in the sterile cover. **Steps F2, F3 are then shifted**. The CT scan focused on the work area is not performed. In this case it is the **initial diagnostic CT scan that is used as the reference for planning the procedure on the navigation system**.

4.2. Primary objective and primary endpoint

Primary objective:

Compare the accuracy ***of local anesthetic needle*** placement under CT guidance in the context of an interventional radiology intervention for the following two practices: intervention using the surgical navigation system (NAV Group - new treatment) and intervention without the surgical navigation system (CT Group - reference treatment).

Details:

We aim to evaluate ***the accuracy of the placement of the local anesthesia needle:***

This is determined for each method independently. The tracking step is common to all procedures and always takes place in an essentially identical manner. During this phase, the operator will be asked to position the local anesthetic needle under the assumption that it represents the definitive trajectory to reach the target. In fact, the local anesthetic needle does not reach the target, but it will be assumed that the trajectory of the needle corresponds to the final trajectory.

Two scenarios are considered:

1. The patient has not moved between the planning phase and the actual carrying out of the intervention: the initial landmarks and the landmarks when the trajectory is determined are unchanged (see Figure 1). The maximum distance *d* between the planned trajectory (blue) and the actual trajectory, defined by the local anesthetic needle (red), is determined in 3D space from the acquired data.

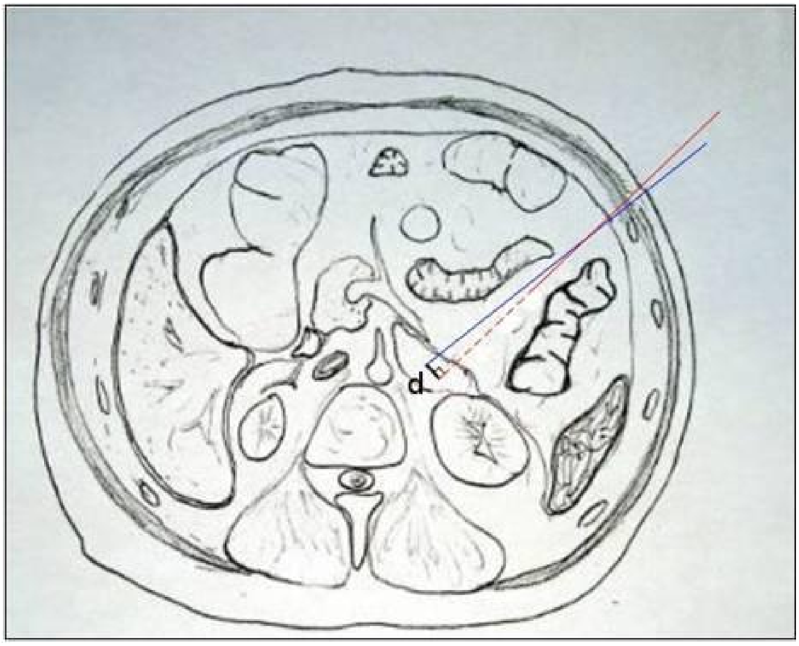

Figure 1 : **maximum distance *d*** *between the planned trajectory (blue) and the tip of the local anesthetic needle extrapolated up to the target (red)*

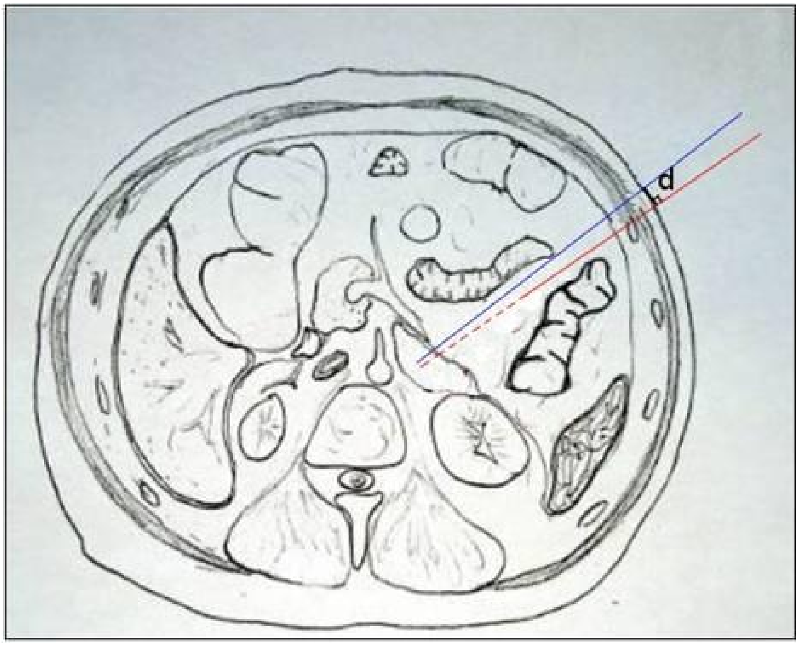

Figure 2: **maximum distance d** *between the planned trajectory (blue) and the tip of the local anesthetic needle extrapolated up to the target (red)*

2. The patient moved between tracking and the actual carrying out of the intervention:
   The landmarks have changed, the target no longer has the same coordinates (Figure 3). This change must be taken into account as a good trajectory may miss the target (red line) and conversely a bad trajectory (green line) could reach it, but passing through organs that must be avoided (such as the colon in this example). The shift in the patient's position will be estimated partly subjectively, and partly objectively by comparing the position of the hybrid localizer in the CT images that were used in the planning step with the control

images. Note: this double estimation will confront the perception of the radiologist with objective reality.

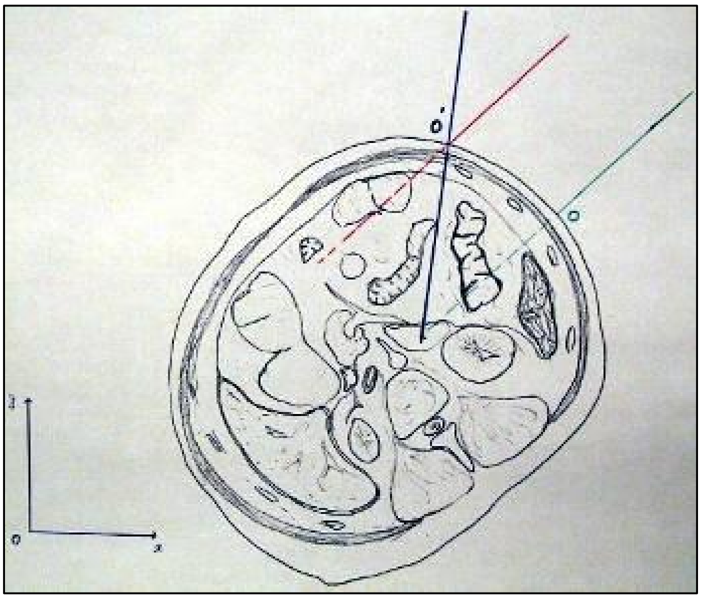

Figure 3: The patient has moved. The coordinates of the target, the entry point and the trajectory are changed in the same coordinate system.

**Primary endpoint (Step 1):**<br>Maximum distance ***d*** between the planned trajectory and the ***extrapolated trajectory of the local anesthesia needle***.<br>The following data will be available:<br>- Planned trajectory: entry point, target point and equation of the line (CT Scanner coordinate system)<br>- Actual trajectory: entry point, target point and equation of the line (CT Scanner coordinate system).<br>These measures concern only the tracking step

For the conventional method, the planned trajectory on the scanner console is compared with the path of the local anesthetic needle obtained on the control CT scan.

With the navigation system, the planned trajectory of the system is compared with the trajectory of the local anesthetic needle obtained on the control CT scan. Thus, the accuracy is determined for each method independently.

4.3. Secondary objectives and secondary endpoints

Objective 1: To compare the accuracy of placement of the puncture needle

The main objective of this study concerned only the tracking phase (placement of the local anesthetic needle) because it is the only step that is essentially identical between the two methods, and this, regardless of the characteristics of the patient or the nature of the intervention made. However, we also

wish to assess the accuracy of placement of the puncture needle. After the tracking phase, the intervention continues with the same technique used for the placement of the local anesthesia needle, which will enable us to compare the accuracy of placement of the final puncture needle between the CT group and the Nav group.

Endpoint 1 (step 2):
- Maximum distance ***d*** between the planned trajectory and the trajectory actually realised by the puncture needle.

The following data will be available:

- Planned trajectory: entry point, target point and equation of the line (CT Scanner coordinate system)
- Actual trajectory: entry point, target point and equation of the line (CT Scanner coordinate system).

Objective 2: Comparison of the duration of the intervention

The comparison of the duration of the intervention between the NAV and CT groups will take into account potential confounders. Temporal data for the different stages of the procedure will be collected:

- H0 : First CT acquisition,
- H1 : Local anesthesia needle in place and control CT scan performed
- H2 : Puncture needle in place and control CT scan performed (first control scan for which the radiologist thinks he/she has reached the target)
- H3 : Final control CT scan for any complications of the intervention

Endpoint 2:

- Duration of the tracking phase (step 1) Δ1 = H1- H0
- Duration of the intervention (step 2) Δ2 = H2- H1
- Total Intervention Time Δ3 = H3-H0

Objective 3: Radiation dose

Compare the total radiation dose between NAV and CT groups

Endpoint 3:

- Dose duration in mGray*cm during Δ1 (from H0 to H1 included)
- Dose duration in mGray*cm during Δ2 (from end H1 to H2 included)
- Total dose duration in mGray*cm (from H0 to H3 included)
- Number of CT slices obtained

Objective 4: Evaluation of the operator's satisfaction

The criteria for operator satisfaction include:

1. The accuracy of the procedure as perceived by the operator

2. Constraints in performing the intervention: for example, the manageability of the tools, constraints related to the tracking device such as positioning the reference marker, setting up the sterile field, artifacts generated by the reference object
3. The interest of each technique when the optimal trajectory plane is not the transverse acquisition plane
4. Freedom of movement
5. The confidence felt by the operator in carrying out his intervention
6. Overall Satisfaction Score concerning how the procedure went and how it was carried out

Each criterion will be assessed using either a visual analog scale or single choice questions.

**Endpoint 4:**
- Overall satisfaction score (NAV group and CT group)

Objective 5: Failure or Success of Intervention

Compare final results (success or failure) of the interventions made with the navigation system and those done with the conventional method. Failures /successes will be defined based on objective data obtained following the completion of the intervention and depending on the indication: drainage under radiological monitoring, pathological outcome of biopsied tissue, etc.

**Endpoint 5:**
- Overall failure or success of the intervention

Objective 6: Number of Attempts

Comparison of the number of attempts to reach the target, for Nav and CT groups. The total number of attempts will be counted, both in the tracking step when positioning the local anesthetic needle and when making the final intervention.

**Endpoint 6:**
- Number of puncture attempts until successful intervention

Objective 7: Complications

Complications that occurred when performing the intervention will be compared between the two groups. We propose to use the SIR (Society of Interventional Radiology) classification used in this field.

TABLE 29.1 SOCIETY OF INTERVENTIONAL RADIOLOGY (SIR) CLASSIFICATION SYSTEM FOR COMPLICATIONS BY OUTCOME

Minor Complications
A. No therapy, no consequence
B. Nominal therapy, no consequence; includes overnight admission for observation only
Major Complications
C. Require therapy, minor hospitalization(<48 hours)
D. Require major therapy, unplanned increase in level of care, prolonged hospitalization (>48 hours)
E. Permanent adverse sequelae
F. Death

Reprinted from Sacks D, McClenny TE, Cardella JF, Lewis CA. Society of Interventional Radiology clinical practice guidelines. *J Vasc Interv Radiol.* 2003;14:S199–S202, with permission from Elsevier.

Endpoint 7:

- Number of immediate and post-interventional complications
- Type of immediate and post-interventional complications (grade A to F of the SIR classification)

Objective 8: Estimation of medical service, expected and rendered (EMR and MSR)

From the different parameters evaluated previously, a first estimate of the expected and potential medical benefit will be carried out.

Objective 9: Consideration of potential confounders

Various potential confounders will be considered during this biomedical research:

- Operator experience (novice / expert)
- Number **n** of navigated interventions already performed by the operator
- Subjective assessment of the accessibility of the target (easy/difficult and/or deep/superficial)
- Interventional Indication
- Patient position (left lateral decubitus, right lateral decubitus, ventral, dorsal)
- Any patient movement (movement/no movement)
- Interventional modality in the navigated group (placement of the hybrid localizers for the tracking step: 1 localizer used and not moved, 1 localizer used and moved, 2 localizers used).

5. RESEARCH STUDY DESIGN

5.1. Study Type

- Clinical trial of innovative medical device

- Single-center study
- Study phase: Equivalent to phase II/III of drug development
- Prospective
- Controlled
- Open
- Parallel group trial
- Randomized

5.2. General organization of the study and flow diagram

5.2.1. Study Patient Flow

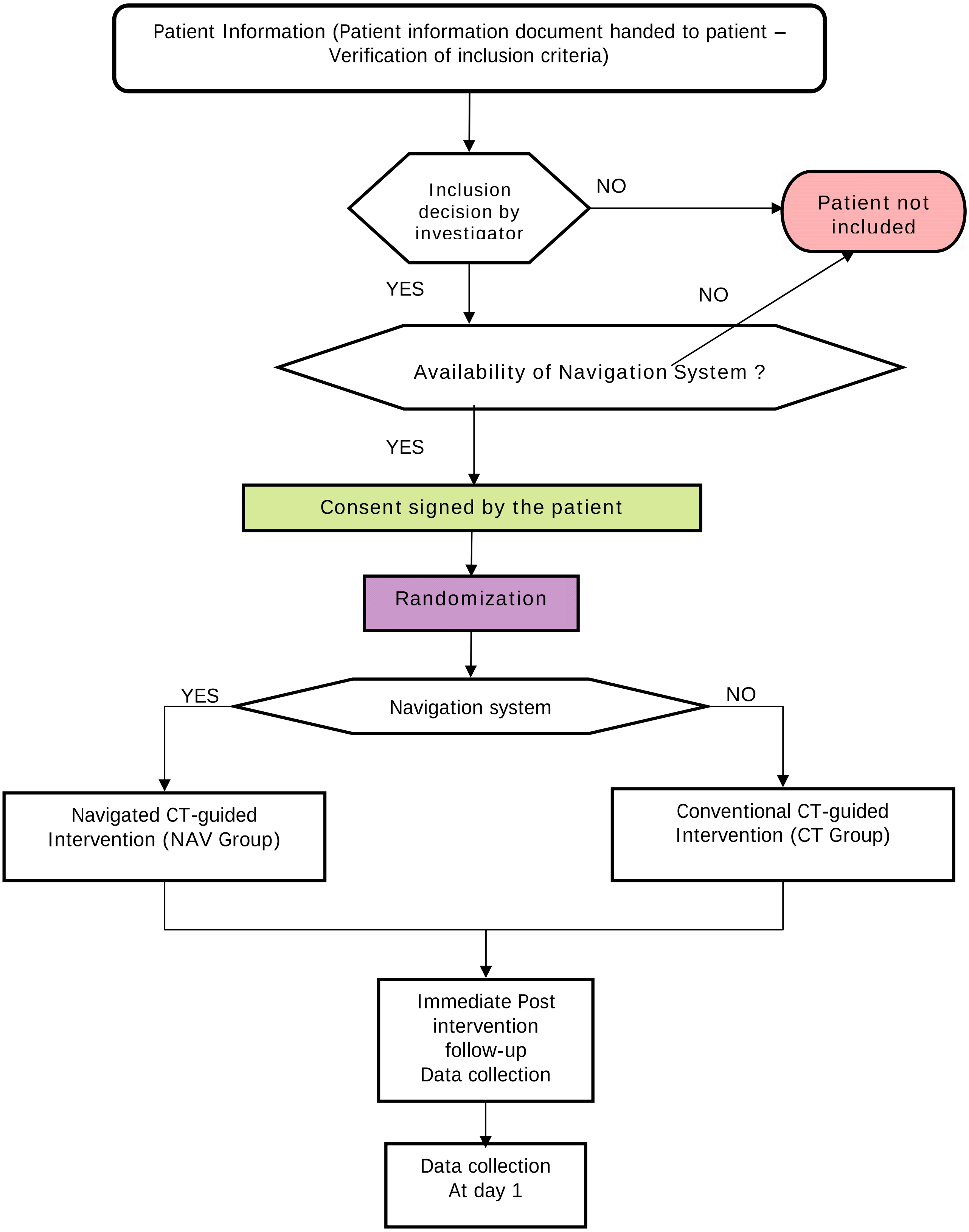

5.2.2. Practical Organization of the study

STAGE 1 - Training of interventional radiologists in use of the navigation system

As use of the medical device under consideration is very intuitive, operators will initially be trained using a phantom (minimum two interventions on the phantom in the presence of the instructor). If the operator wants to have more training so as to be more comfortable with the system, he will be able to do so. Inclusions can begin following this training. To prevent a possible difficulty with the first use of the new device on a patient, the first two interventions will be carried out in the presence of the radiologist instructor. According to the level of ease felt by the operator, the next interventions will be conducted autonomously.

STAGE 2 - Recruitment of patients in the radiology department

The recruitment of potential patients will take place when the treatment plan is being decided. Once the decision has been made, an information form will be given to potentially eligible patients. Following a period of reflection, if he/she wishes to participate in the study, the patient will sign the consent form. A copy of these documents will be given to the patient.

Before any inclusion, the availability of the navigation system will be verified.

STAGE 3 – Realization of the intervention

The patient, having thus agreed to participate in this biomedical research will receive "normal" care in the radiology department as part of the protocol. They will be randomized into one of the two groups (CT, Nav), the intervention will be carried out either conventionally (CT group) or guided by the navigation system (Nav group). With the conventional method, the introduction of instruments will be done under scanner control only, while with the navigation system, the operator uses the information provided by the system and will also have the possibility to check the progress of the needle into the body by performing CT scans. During the tracking phase, the operator will have planned the introduction of the needle and will have introduced it in accordance with this planned trajectory. The operator will then use again this trajectory to continue the procedure so as to achieve the desired intervention using the initial guidance mode (conventional /navigated).

Of course, at any time, if desired, the patient may withdraw consent and no longer participate in this biomedical research.

STAGE 4 – Patient Monitoring in immediate post-interventional

During the usual post-interventional monitoring of the patient, the radiologist will record the patient's demographic and clinical characteristics in the electronic CRF.

STAGE 5 - Clinical evaluation of the patient during follow-up visit

Patients who had an intervention will be evaluated during a follow-up visit.

5.2.3. Investigation procedures different from usual care

This study does not involve any differences in patient care

5.3. Study Site

Grenoble University Hospital (CHU Grenoble): Radiology and Medical Imaging University Clinic (**CURIM**)

.

6. CHARACTERISTICS OF SUBJECTS

6.1. Subject recruitment

Patients attending the study center for whom an indication for an intervention has been decided by consensus following a discussion between radiologists, clinicians and surgeons, will be recruited.

6.2. Inclusion criteria

Subjects meeting all the following criteria will be proposed the study:

•Adult,

• Person affiliated to the French social security or beneficiary of an equivalent scheme,

• Person for whom a diagnostic or therapeutic interventional percutaneous CT-guided intervention is indicated. This intervention will be carried out after a clinical examination whose findings will be communicated directly to the patient.

6.3. Non-inclusion criteria

Subjects meeting at least one of the following criteria will not be included:

• Pregnant women, women in labor, and nursing mothers,

• Persons deprived of liberty by judicial or administrative decision,

• People hospitalized without consent (compulsory hospitalization or at the request of a third party),

• Adults subject to legal protection or unable to consent,

• Any of the usual contraindications for interventional radiology

• Any of the usual contraindications for MRI. In particular, the use of the navigation system will be excluded for patients with non-MRI compatible equipment such as a pacemaker.

• Also excluded are patients with intracorporeal ferromagnetic foreign bodies near the radiologist’s work area and which may interact with the system, even if these foreign bodies are not a contra-indication for MRI.

6.4. Concomitant treatments

Once the interventional indication has been decided by consensus, all treatments that are not contra-indicated to the intervention are allowed.

7. VARIABLES TO BE MEASURED AND METHODS

7.1. Clinical parameters

The main clinical parameters recorded are the following:

- Sex,
- Age

7.2. Para-clinical parameters

The following parameters will be recorded:

- Coagulation parameters: PR, APTT, INR, Platelet count
- Interventional indication,
- The accessibility of the target,
- Position the patient during surgery,
- Operator experience (novice/expert)
- The number n of navigated interventions already performed by the operator
- The precision of the intervention tracking phase (comparison of planned and actual trajectories for the local anesthesia needle)
- Precision of the intervention during the procedure (comparison of planned and actual trajectories for the puncture needle)
- Duration of the intervention (tracking phase and total)
- The irradiation dose (dose x duration)
- The success of the intervention (pathology results)
- The number of puncture attempts until success
- Interventional complications
- Qualitative and comparative assessment of the new technique (evaluation questionnaire)
- Limitations encountered with the use of the navigation system (evaluation questionnaire)
- Movement of the patient
- Interventional modality in the navigated group (hybrid localizer).

7.3. Data collected directly in the case report form

Apart from the clinical examination required as part of the study, all of the data described above will be entered directly into the study's electronic case report form.

8. TREATMENT OR PROCEDURES TO BE COMPARED

8.1. Test treatment

The "treatment" to be tested is the use of the navigation system during a diagnostic or therapeutic intervention, under CT guidance.

8.2. Reference treatment

The "reference treatment" is the conventional process for diagnostic or therapeutic interventions under CT guidance.

8.3. Random allocation method (centralized randomization)

After verification of the criteria for inclusion and non-inclusion, centralized randomization will be done electronically by an independent service company external to the CHU. In particular, a randomization site will be available to investigators 24h / 24, in order to have a tool adapted to the lifestyle of radiologists.

9. DATA COLLECTION AND MANAGEMENT

9.1. Data collection

All data from the interrogation and clinical examination should be available in a specific study folder, available to the sponsor, which will constitute the source data.
The case report forms will be filled out and signed by the investigator.
In agreeing to participate, the investigator agrees to conduct research in strict accordance with the experimental protocol, the principles of "Good Clinical Practice" and the legislative and regulatory provisions in force. He vouches for the authenticity of the data collected in the context of the study and accepts the legal provisions authorizing the study sponsor to implement a quality control.
Statistical analysis will be performed only after checking the data entered and the coherence of the data. The data will be archived by the coordinator.

9.2. Monitoring

Data monitoring will be conducted on all data by a Clinical Research Associate or an engineer from the CIC-IT of CHU Grenoble. It will cover 10% of report forms drawn at random.
All patient consent forms will be checked, as well as the inclusion/non-inclusion criteria, and adverse events.
A report of the monitoring will be prepared by the CRA and/or engineer and kept in the study folder.

10. STATISTICAL ANALYSIS

10.1. Steering committee

A steering committee will be formed at the beginning of the study. It will include Dr. Ivan Bricault, Dr. Alexandre Moreau-Gaudry and Prof. Jean-Luc Bosson. This committee will decide, in particular with respect to the results obtained from the interim analysis, as to whether the study should continue or be stopped. It will also be responsible for the management of any changes to the original statistical plan.

10.2. Data analysis strategy

10.2.1. Description of the study population

Quantitative parameters will be described by their mean ± standard deviation, median [25% percentile - 75% percentile], maximum and minimum. Qualitative parameters will be expressed in number and percentage with the 95% confidence interval.
A comparison of the 2 study groups (NAV and CT) will be conducted to check the proper implementation of randomization and describe any potential confounders.

10.2.2. Choice of type 1 error (~) and power

Analysis of the endpoints will be comprehensive (CT vs NAV). As is usual practice, the risk of an α type I error is fixed at 5%, with a power of 90%.

An interim analysis is planned one year after the date of the first included patient, to ensure the proper proceeding of the study (with, in particular, a first evaluation of the navigation system and its compatibility with its specifications, as well as an assessment the actual recruitment compared to the expected recruitment). To maintain an overall threshold of 5% at the final analysis, a 0.1% threshold will be retained for the interim analysis [19]

10.2.3. Description of endpoints to be studied

Primary Objective

According to data obtained in preclinical studies (see Appendix A), the average distance between the end of the needle and the target in the Nav group is significantly different from that in the CT group. This biomedical research has therefore been designed as a bilateral comparison test of the means of the Nav versus CT groups according to the distance criterion.

The analysis will be carried out as intention to treat. It may be accompanied by a per protocol analysis. Given the absence of a double-blind aspect of this study, a posteriori adjustment methods (multiple linear modeling) will allow adjustment of the variable in order to explain the previously identified potential confounders.

Secondary Objectives

Each endpoint will be the object of a univariate analysis comparing the 2 groups studied. The Student t test will be used to compare quantitative variables. In the event that the conditions for this test are not met, non-parametric tests will be used. Potential links between qualitative parameters will be objectified using the chi-square test or Fisher's exact test if numbers are insufficient. Then, given the absence of double-blinding, *a posteriori* adjustment methods (multiple linear modeling) will be implemented to adjust the results according to potential confounders.

The learning curves for the various operators will be represented graphically.

10.3. Calculation of Number of subjets

The calculation of the number of subjects is based on the validation of the main objective according to the preclinical data results. Using the usual methods of calculation the following assumptions were used (see Appendix A):

- $\mu_{CT} = 13$
- $\mu_{Nav} = 6$
- $\sigma = Max(11.3 ; 4) = 11.3$
- $\alpha = 0.05$
- power = 0.9

Thus the number of subjects needed is 56 per group. The theoretical total number of patients to include is therefore 112. Assuming a potential 5% lost to follow-up, then the effective number of subjects to include is 59 per group, approximately 120 in total.

10.4. Responsability for analyses

The person responsible for the statistical analyses will be one of the following people at Grenoble University Hospital:

- Doctor Ivan Bricault, Lecturer, Hospital Practitioner at CURIM,
- Doctor Alexandre Moreau-Gaudry, Hospital/University assistant professor at CIC-IT and the Department of Methodology and Information in Health (DMIS),
- Mr Jean-Louis Quesada, biostatistician at the CIC, Grenoble University Hospital.

10.5. Site of data analyses and software

The CURIM or the CIC-IT at Grenoble will be the site of analysis of the anonymized data. This analysis will be made after freezing the database and will respect good practices for statistical analysis. It will be performed using GNU-R software.

REFERENCES

1. General Principles for Evaluation of New Interventional Technologies and Devices. Sacks D, Marinelli D, Martin L et al. J Vasc Interv Radiol 2003 ;14 :S391-S394

2. Quality Improvement Guidelines for Image-guided Percutaneous Biopsy in Adults. Cardella JF, Bakal CW, Bertino RE et al. J Vasc Interv Radiol 2003 ;14 :S227-S230

3. Quality Improvement Guidelines for Adult Percutaneous Abscess and Fluid Drainage

Bakal CW, Sacks CD, Burke DR et al. J Vasc Interv Radiol 2003 ;14 :S223-S225

4. Society of Interventional Radiology Quality Improvement Guidelines for Percutaneous Vertebroplasty. McGraw JK, Cardella J, Barr JD et al. J Vasc Interv Radiol 2003 ;14 :S311-S315

5. CT-guided procedures: evaluation of a phantom system to teach accurate needle placement. Dimmick S, Jones M, Challen J Clin Radiol. 2007 Feb;62(2):166-71.

6. CT-guided percutaneous transthoracic biopsy in the evaluation of undetermined pulmonary lesions. Lourenco R, Camacho R, Barata MJ Rev Port Pneumol. 2006 Sep-Oct;12(5):503-24

7. CT-guided bone biopsy: Initial experience with a commercially available hand held Black and Decker drill. Buckley O, Benfayed W, Geoghegan T. Eur J Radiol. 2007 Jan;61(1):176-80.

8. Augmented reality visualization for CT-guided interventions: system description, feasibility, and initial evaluation in an abdominal phantom. Das M, Sauer F, Schoepf UJ, Radiology. 2006 Jul;240(1):230-5.

9. Accuracy and complications in computed tomography fluoroscopy-guided needle biopsies of lung masses. Heck SL, Blom P, Berstad A. Eur Radiol. 2006 Jun;16(6):1387-92.

10. Out-of-plane computed-tomography-guided biopsy using a magnetic-field-based navigation system. Wallace MJ, Gupta S, Hicks ME. Cardiovasc Intervent Radiol. 2006 Jan-Feb;29(1):108-13

11. Computed-tomography-guided punctures using a new guidance device. Magnusson A, Radecka E, Lonnemark M et al. Acta Radiol. 2005 Aug;46(5):505-9.

12. Complications of fluoroscopy-, ultrasound-, and CT-guided percutaneous interventional procedures. Dupas B, Frampas E, Leaute F, J Radiol. 2005 May;86:586-98.

13. Design and analysis of randomized clinical trials requiring prolonged observation of each patient. I. Introduction and design. Peto R, Pike MC, Armitage P et al. Br J Cancer. 1976 Dec;34(6):585-612.

14. Electromagnetic tracking for abdominal interventions in computer aided surgery. Zhang H, Banovac F, Lin R et al. Comput Aided Surg. 2006 May;11(3):127-36.

15. Intérêt de l'utilisation d'une station de navigation dans le guidage des gestes effectués sous tomodensitométrie. Bing F, Bricault I, Sengel C, Ferretti G. In: Proceedings of the Third International Conference Surgetica'2007, Chambéry. Merloz P, Troccaz J, editors ; Sauramps Médical, 2007.

16. Needle biopsy of anatomically unfavorable liver lesions with an electromagnetic navigation assist device in a computed tomography environment. Banovac F, Wilson E, Zhang Het al. J Vasc Interv Radiol. 2006 Oct;17(10):1671-5.

17. Value of a laser guidance system for CT interventions: a phantom study. Jacobi V, Thalhammer A, Kirchner J. Eur Radiol. 1999;9(1):137-40.

18. A Navigation System for Minimally Invasive CT-Guided Interventions. Nagel M, Schmidt G, Petzold Ret al. Gerig Editors; MICCAI 2005, LNCS 3750, Springer-Verlag, pp. 33–40, 2005.

19. Peto R, Pike MC, Armitage P, Breslow NE, Cox DR, Howard SV, Mantel N, McPherson K, Peto J, Smith PG. Design and analysis of randomized clinical trials requiring prolonged observation of each patient. I. Introduction and design. Br J Cancer. 1976 Dec;34(6):585-612.

APPENDIX A: Preclinical Results – Primary Endpoint

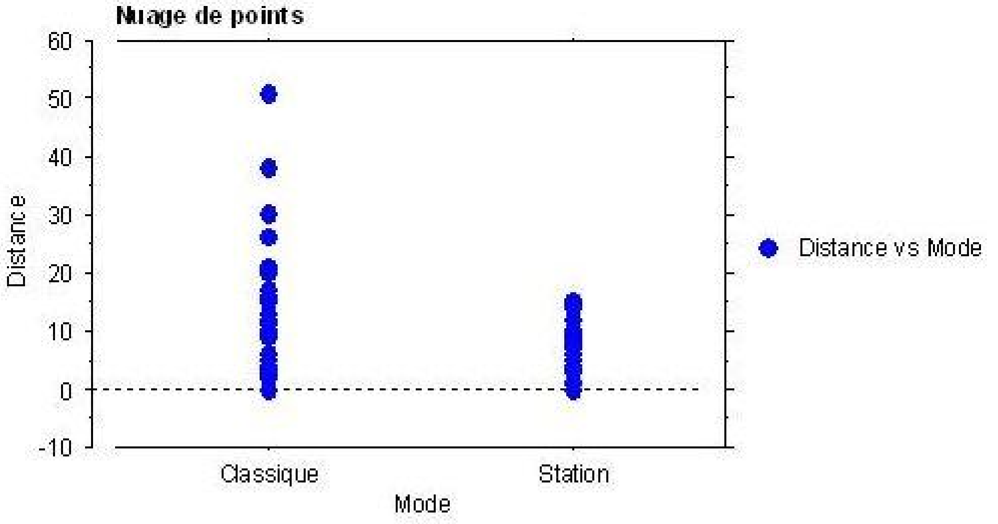

Statistiques descriptives

	Distance
Moy.	9,444
Dév. Std	9,125
Erreur Std	1,075
Nombre	72
Minimum	0,000
Maximum	51,000
# Manquants	0

Test-t séries non appariées pour Distance
Variable "groupe" : Mode
Ecart théorique = 0

	Ecart moyen	DDL	t	p
Classique, Station	7,056	70	3,536	,0007

Info. du groupe pour Distance
Variable "groupe" : Mode

	nombre	Moy.	Variance	Dév Std	Erreur Std
Classique	36	12,972	127,513	11,292	1,882
Station	36	5,917	15,793	3,974	,662

Moy. = mean ; Dév Std. = standard deviation ; Erreur Std. = standard error ; Manquants = missing values

Station = navigation system ; Classique = conventional
