# Supplementary material for: Computer assisted electromagnetic navigation improves accuracy in computed tomography guided interventions: A prospective randomized clinical trial
Source: PLoS One. 2017 Mar 15;12(3):e0173751. doi: 10.1371/journal.pone.0173751 (PMC5351986; doi:10.1371/journal.pone.0173751)
Supplement: S2 File — (PDF) [file pone.0173751.s002.pdf]

Pôle d'imagerie

PROTOCOLE EXPERIMENTAL

1. INFORMATIONS GENERALES

Titre : Intérêt de l'utilisation d'une station de navigation dans le guidage des gestes effectués sous tomodensitométrie
Titre court : CT-Nav

**N°d'enregistrement AFSSAPS : 2008-A00742-53**	**N° attribué par le promoteur : 0812**
**Date : 6 juillet 2010**	**Version : 11.4**

Méthodologie : Essai clinique monocentrique, prospectif, en groupes parallèles, randomisé, en ouvert, contrôlé

Phase d'étude : Equivalent de phase II/III médicamenteuse

Dispositif médical à l'étude : Station de navigation pour la ponction sous scanner

Investigateur coordonnateur autorisé à signer le protocole : Dr I. Bricault (GRENOBLE)
Investigateur coordonnateur : Dr I. Bricault (GRENOBLE)

**Investigateurs :**<br>Pr G. Ferretti<br>Dr C. Sengel	Dr JN. Ravey<br>Dr M. Rodière<br>Dr A. Jankovski	Dr L. Felix<br>Dr V. Cuvinciuc<br>Dr C. Dubois<br>Dr O. Eker

Participants : internes de la Clinique Universitaire de Radiologie et d'Imagerie Médicale du CHU de Grenoble

Intervenants : Dr A. Moreau-Gaudry (Centre d'Investigation Clinique - Innovation Technologique, CIC-IT), M. Lionel Carrat (Société Imactis), Mlle K. Guerrero (CIC-IT), Mlle AS Silvent (CIC-IT)

Centre d'étude : C.H.U. de Grenoble – Pole d'imagerie – Clinique Universitaire de Radiologie et d'Imagerie Médicale (CURIM)

Promoteur : C.H.U. de Grenoble
Représentant du promoteur autorisé à signer le protocole : Frédérick Marie
Direction de la Recherche Clinique et à l'Innovation, CHU de Grenoble, 38043 Grenoble Cedex 09
Tel : 04 76 76 59 57 Fax : 04 76 76 52 21

Centre coordonnateur de l'étude :
Centre d'Investigation Clinique – Innovation Technologique– Inserm 803
CHU de Grenoble, 38043 Grenoble Cedex 09
Tel : 04 76 76 92 60 Fax : 04 76 76 92 62

Avis favorable du CPP Sud-Est V, le : 01/07/2009

SOMMAIRE

1. INFORMATIONS GENERALES 1

2. ABREVIATIONS ET DEFINITIONS 3

2.1. ABRÉVIATIONS 3

3. JUSTIFICATION DE L'ÉTUDE 3

3.1. PRÉSENTATION GÉNÉRALE : 3
3.2. DESCRIPTION GÉNÉRALE DU DISPOSITIF MÉDICAL 4
3.3. RÉSULTATS DES ESSAIS PERTINENTS AU REGARD DE LA RECHERCHE BIOMÉDICALE 6

4. OBJECTIF(S) DE L'ÉTUDE 6

4.1. PRÉSENTATION ET OBJECTIF GÉNÉRAL 6
4.2. OBJECTIF PRINCIPAL ET CRITÈRE DE JUGEMENT PRINCIPAL 9
4.3. OBJECTIFS SECONDAIRES ET CRITÈRES DE JUGEMENT SECONDAIRES 11

5. CONCEPTION DE LA RECHERCHE 15

5.1. TYPE D'ÉTUDE 15
5.2. ORGANISATION GÉNÉRALE DE L'ÉTUDE ET SCHÉMA 15
5.3. LIEUX DE L'ÉTUDE 17

6. CARACTERISTIQUES DES SUJETS 17

6.1. MODALITÉS DE RECRUTEMENT DES SUJETS 17
6.2. CRITÈRES D'INCLUSION 18
6.3. CRITÈRES DE NON-INCLUSION 18
6.4. TRAITEMENTS ASSOCIÉS AUTORISÉS 18

7. VARIABLES MESURÉES ET MÉTHODES DE MESURE 18

7.1. PARAMÈTRES CLINIQUES 18
7.2. PARAMÈTRES PARA-CLINIQUES 18
7.3. DONNÉES RECUEILLIES DIRECTEMENT DANS LE CAHIER D'OBSERVATION 19

8. TRAITEMENT OU PROCEDURES COMPAREES 19

8.1. TRAITEMENT TESTÉ 19
8.2. TRAITEMENT DE RÉFÉRENCE 19
8.3. MÉTHODE D'ALLOCATION ALÉATOIRE (RANDOMISATION CENTRALISÉE) 19

9. RECUEIL ET GESTION DES DONNEES 19

9.1. RECUEIL DES DONNÉES 19
9.2. MONITORAGE 20

10. ANALYSE STATISTIQUE DES PARAMÈTRES MESURÉS 20

10.1. COMITÉ DE PILOTAGE 20
10.2. STRATÉGIE D'ANALYSE DES DONNÉES 20
10.3. CALCUL DU NOMBRE DE SUJETS 21
10.4. RESPONSABLE DES ANALYSES 21
10.5. LIEU D'ANALYSE DES DONNÉES ET LOGICIELS UTILISÉS 22

RÉFÉRENCES BIBLIOGRAPHIQUES 23

2. ABREVIATIONS ET DEFINITIONS

2.1. Abréviations

**ARC**	:	**A**ttaché de **R**echerche **C**linique
**CHU**	:	**C**entre **H**ospitalier et **U**nversitaire
**CIC-IT**	:	**C**entre d'**I**nvestigation **C**linique – **I**nnovation **T**echnologique
**CPP**	:	**C**omité de **P**rotection des **P**ersonnes
**CURIM**	:	**C**linique **U**niversitaire de **R**adiologie et d'**I**magerie **M**édicale
**INR**	:	**I**nternational **N**ormalized **R**atio
**HO**	:	**H**ospitalisation d'**O**ffice
**TCA**	:	**T**emps de **C**éphaline **A**ctive
**TDM**	:	**T**omo**D**ensito**M**étrie
**TP**	:	**T**aux de **P**rothrombine

3. JUSTIFICATION DE L'ÉTUDE

3.1. Présentation générale :

3.1.1. Problématique

Initialement utilisés en mode scopie et par la suite en tomodensitométrie, les rayons X sont une aide précieuse au guidage des gestes effectués par abord percutané en radiologie interventionnelle. Si certains gestes peuvent bénéficier d'un simple guidage échographique, de nombreuses lésions inaccessibles ou peu échogènes nécessitent le recours à un guidage tomodensitométrique. L'objectif de ces gestes est soit diagnostique (analyse histologique ou bactériologique) soit thérapeutique (infiltrations foraminales, drainage des abcès pleuraux, traitement des lésions tumorales par radiofréquence, autres).

Le dénominateur commun à toutes ces interventions est l'étape préalable d'anesthésie locale, qui consiste à guider une aiguille d'anesthésie locale jusqu'à une cible sous-cutanée.

L'examen scanner fournit un ensemble de coupes transversales : il est alors aisé de planifier une trajectoire dans le plan de coupe transverse. L'opération se complique lorsqu'il s'agit de planifier une trajectoire oblique par rapport à ce plan du fait de la présence de structures nobles (artères, organes à risques) ou d'obstacles (os) puisque cela impose l'analyse de reconstructions multi-planaires des coupes TDM. De surcroît, la réalisation effective de ce geste est rendue difficile par la nécessité d'estimer des angulations complexes et de visualiser le trajet de son aiguille à l'aide d'une reconstruction et d'une projection « mentale » de l'anatomie du patient. Dans certains cas, du fait de cette complexité, l'opérateur est contraint de choisir une trajectoire dans le plan transverse, plutôt qu'une trajectoire oblique, bien que celle-ci ne soit pas optimale. Par ailleurs, plus la réalisation du geste planifié est difficile, plus les acquisitions TDM de contrôles intermédiaires sont multipliées au prix d'une augmentation de la dose totale d'irradiation pour le patient.

La station de navigation permet d'obtenir, à partir des données d'acquisition du scanner, une vision 3D de l'anatomie du patient et de superposer sur celle-ci, en **temps réel**, le trajet de l'aiguille de ponction.

L'aiguille de ponction est équipée d'un récepteur magnétique repéré en 3D à l'aide d'un émetteur magnétique porté par le référentiel hybride. L'opérateur visualise ainsi directement sur un écran la trajectoire de l'aiguille, aussi bien pendant la phase de planification du geste (effectuée de façon simple en déplaçant l'aiguille au-dessus du patient sans pénétrer la peau, et en cherchant ainsi la trajectoire optimale) que pendant la réalisation de la ponction proprement dite. Il bénéficie d'une représentation de la trajectoire selon trois plans de l'espace alignés en permanence avec la position de l'aiguille, et donc éventuellement obliques. Il s'affranchit ainsi des contraintes de la procédure classique qui l'incitaient à rester dans le plan transversal d'acquisition. De plus, l'opérateur bénéficie d'un contrôle de son geste en temps réel sans impliquer d'irradiation.

Au regard des problèmes précédemment énoncés, l'utilisation de la station de navigation devrait permettre :

1- De multiplier les possibilités de trajectoire
2- D'augmenter la précision du geste
3- De diminuer la durée du geste
4- De diminuer la pénibilité ressentie par le patient
5- De diminuer la dose totale d'irradiation

L'objet de cette étude est d'évaluer l'apport d'un système de navigation récemment développé aux problèmes précédemment identifiés relativement au guidage de l'aiguille.

3.1.2. Les données de la littérature

A notre connaissance, il n'existe pas de données de la littérature rapportant des études cliniques relatives au guidage d'une aiguille lors d'une procédure de radiologie interventionnelle associant Tomodensitométrie et système de navigation.

Les systèmes de navigation sont déjà utilisés en chirurgie orthopédique et urologique. Ils sont d'une aide précieuse pour le chirurgien qui peut avancer une aiguille ou un trocart dans le corps du patient tout en ayant une visualisation précise des structures anatomiques. Des dispositifs informatisés d'aide à la biopsie ont également été évalués avec d'autres techniques d'imagerie comme l'échographie et la fluoroscopie, notamment dans la ponction rénale percutanée.

L'alternative séduisante en radiologie interventionnelle tomodensitométrique est l'utilisation de la fluoro-TDM. Cet outil permet de contrôler l'avancée de l'aiguille en effectuant des rayons X. Le radiologue peut soit effectuer un contrôle continu donc dynamique de l'avancée de l'aiguille, soit faire des contrôles rapides et ponctuels après mobilisation de l'aiguille. Dans ces deux situations, il existe une irradiation supplémentaire à la fois pour le patient et le radiologue.

3.2. Description générale du Dispositif Médical

3.2.1. Identification du dispositif

Le dispositif médical, non marqué CE, illustré au paragraphe 3.1.1, et utilisé dans cette recherche biomédicale est composé de :

1- La station de navigation
2- Le logiciel de navigation spécifique pour la ponction percutanée sous contrôle TDM.

3- Un ancillaire de navigation comprenant :

   a. Le porte-aiguille en plastique stérilisable
   b. Le référentiel hybride en plastique équipé de l'émetteur magnétique.
   c. Des housses stériles à usage unique pour encapsuler les émetteurs et récepteurs magnétiques
   d. Une clé USB fournie à chaque intervention afin de lancer le logiciel et de stocker transitoirement les données nécessaires à la réalisation de la Recherche Biomédicale.

3.2.2. Identification du fabricant

Ce dispositif est fabriqué par la Société IMACTIS située au 5, avenue du grand Sablon, 38700 LA TRONCHE, numéro SIRET 509 967 030 00016 , téléphone 06 60 50 25 24, email : info@imactis.com, site web http://www.imactis.com.

3.2.3. Instruction d'utilisation et installation

La station de navigation est composée d'un chariot mobile équipé d'un PC, d'un écran tactile et d'un système de localisation magnétique. Elle est placée dans la salle de radiologie lors de l'intervention comme illustré dans l'image ci-dessous. Pour utiliser la station de navigation des instruments sont fournis par IMACTIS®

- Porte-aiguille : Instrument stérilisable
- Référentiel hybride : Instrument non stérilisable. Ce dispositif peut être inséré dans une housse stérile à usage unique classiquement utilisée pour toute instrumentation non stérilisable.

<table>
<tr><td>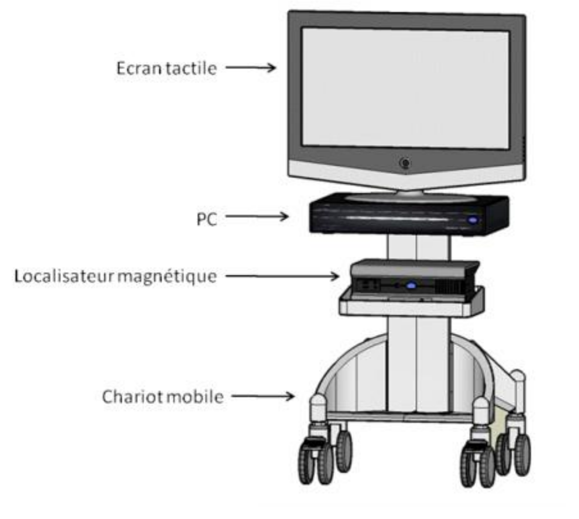
</td><td>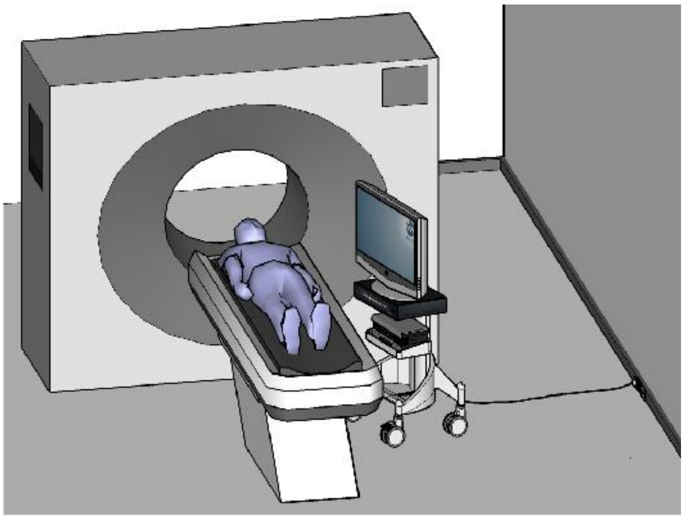</td></tr>
<tr><td>Station de navigation</td><td>Station de navigation dans la salle d'intervention</td></tr>
</table>

Le principe de fonctionnement repose sur le repérage dans l'espace et en temps réel, à l'aide d'un localisateur magnétique, de la position du porte-aiguille par rapport au patient. Par l'intermédiaire du recalage, le scanner du patient pourra être replacé sur l'anatomie réelle du patient. En conclusion, le radiologue pourra visualiser en temps réel la position de son instrument dans les images scanner du patient, comme illustré dans l'image ci-dessous.

Ce principe est déjà utilisé en routine clinique par les orthopédistes, les ORL et les neurochirurgiens,

3.3. Résultats des essais pertinents au regard de la recherche biomédicale

Une évaluation préliminaire de la station avec le système de localisation optique a été effectuée sur fantôme en salle de radiologie interventionnelle. Il s'agissait de s'exercer sur un fantôme, constitué d'une mousse collée sur une planche de bois, elle-même percée de plusieurs trous aux diamètres et inclinaisons variables. Le radiologue devait tenter de traverser la mousse et se positionner au niveau des trous en condition tomodensitométrique. Deux types de ponction ont été réalisés : les premières, de manière conventionnelle sans aide de la station de navigation, les secondes, à l'aide de la station. 36 ponctions ont été réalisées dans chaque groupe par des opérateurs différents. Le critère principal observé était la distance de l'extrémité de l'aiguille par rapport à la cible. Cette distance a été mesurée sur l'image TDM de contrôle. Les critères secondaires explorés étaient relatifs aux temps d'intervention selon les différentes modalités (navigué/ non navigué), aux angulations par rapport aux plans axial et sagittal.

Cette première étape avait pour but:
- de vérifier dans des conditions idéales (objet inerte) l'exactitude des images fournies par la station de navigation en les comparant à celles du scanner;
- de se familiariser avec la station;
- de comparer la précision de chacune des méthodes.

Les premiers résultats ont montré, de manière significative, une meilleure précision en termes de distance par rapport à la cible (cf. Annexe A), d'angulation dans le plan axial transverse ainsi qu'un gain de temps avec l'utilisation de la station.
Une évaluation préliminaire de la station avec le localisateur magnétique sera également conduite dans les mêmes conditions pour confirmer que le système magnétique est équivalent au système optique et apporte les mêmes bénéfices. Cette évaluation sera naturellement jointe au dossier technique et sera adressée aux autorités compétentes.

4. OBJECTIF(S) DE L'ÉTUDE

4.1. Présentation et objectif général

Conformément à la pratique courante, tous les patients candidats à l'étude auront bénéficié au préalable d'une imagerie (échographique, IRM ou scanographique) ayant mis en évidence une lésion nécessitant un geste interventionnel, diagnostique ou thérapeutique, sous scanner. L'indication du geste a fait l'objet d'une discussion entre radiologues, cliniciens et chirurgiens et se déroule de la façon suivante :

- **La position** (décubitus dorsal, ventral ou latéral) du patient sur la table est déterminée par le radiologue qui se réfère au premier bilan d'imagerie. Elle dépend essentiellement de la localisation de la cible. On tiendra compte de la tolérance du patient à cette position.
- **L'étape de repérage** (étape 1) débute par la première acquisition TDM et se termine après la mise en place de l'aiguille d'anesthésie locale. **Dans le cadre de cette étude, l'opérateur sera informé de la nécessité de grande précision de sa trajectoire d'anesthésie locale au cours de la phase de repérage puisque celle-ci représentera par extrapolation la trajectoire définitive voulue.**

- **Le geste interventionnel** (étape 2) est réalisé après l'anesthésie locale et consiste à atteindre, avec une aiguille, la lésion ou l'organe cible qui fait l'objet de l'intervention.

Cette étude a pour objectif l'étude de la trajectoire de ponction réalisée soit avec la méthode classique soit avec la station de navigation. Chaque patient bénéficiera, de manière aléatoire, soit de l'une soit de l'autre des méthodes, du début à la fin du geste. Nous estimons que **seule l'étape de repérage est globalement identique entre les deux méthodes, et ceci, indépendamment du patient ou du geste interventionnel réalisé**. Pour cette raison nous définirons le critère de jugement principal uniquement sur cette étape de repérage.

4.1.1. La procédure classique (Groupe CT)

Avant l'acquisition scanographique, un objet plastique (identique à celui utilisé dans la procédure de navigation : le référentiel hybride) est placé sur le patient. Il permet la définition d'un repère à partir duquel des mesures sont réalisées pour préparer le geste interventionnel. L'opérateur définit sur la console du scanner la trajectoire optimale qu'il souhaite obtenir : c'est la planification qui servira de référence *(**trajectoire planifiée**)*. Cette trajectoire peut ne pas être dans le plan de l'acquisition et nécessiter la réalisation de reconstructions. L(es)'image(s) qui servira(ont) de **référence** est(seront) **annotée(s)** avec identification du point d'entrée et du point cible. Cette(s) image(s) est(seront) alors **enregistrée(s)**.
Le niveau de coupe intéressant le trajet et le point d'entrée cutanée est noté sur la console scanner. Le manipulateur matérialise le point d'entrée au feutre sur la peau en s'aidant du repérage scanographique indiquant le niveau de coupe et en mesurant la distance objet métallique - point d'entrée. Le radiologue, sans aide visuelle, met en place l'aiguille d'anesthésie locale après avoir déterminé avec précision le point d'entrée et l'angle de pénétration de l'aiguille. Une fois l'aiguille en place, on réalise le premier contrôle scanographique. La position de l'aiguille d'anesthésie locale dans les images scanographiques indique la ***trajectoire réalisée*** (point d'entrée et point cible). Les trajectoires **planifiées** et **réalisées seront alors comparées**.

Une fois l'étape de repérage terminée, le geste se poursuit par la ponction avec la même technique utilisée pour la mise en place de l'aiguille d'anesthésie locale. Une fois la ponction réalisée, un scanner de contrôle est effectué. La position finale de l'aiguille de ponction dans les images scanographiques matérialise la trajectoire réalisée.

4.1.2. La nouvelle procédure (Groupe NAV)

Les étapes du protocole opératoire sont décrites de manière schématique ci-dessous.

A - Préparation de la station de navigation
1/ La station de navigation est allumée et démarre automatiquement
2/ Le logiciel de navigation est lancé par l'intermédiaire de la clé USB
3/ Le logiciel vérifie la présence de la connexion réseau informatique du CHU et du branchement des composants magnétiques
4/ Une fois la vérification précédente effectuée, le logiciel est en attente de réception d'un examen scanner.

B - Préparation de l'instrumentation:

1/ Le référentiel hybride équipé de l'émetteur magnétique est sorti de son emballage, prêt à être collé au voisinage de la région à ponctionner.
2/ Un capteur magnétique est fixé sur le porte-aiguille non stérile
3/ Un capteur magnétique est inséré dans la housse stérile puis fixé sur le porte-aiguille stérile.

C - Installation du patient :
1/ Protocole habituel
2/ En fin d'installation, le référentiel hybride équipé de l'émetteur magnétique est fixé sur la peau du patient au voisinage de la région à ponctionner.

D - Réalisation du scanner de diagnostic :
1/ Selon protocole habituel
2/ En fin d'acquisition scanner, l'examen est « poussé » sur la station de navigation (en attente de cet examen) par le réseau informatique de l'hôpital en suivant le protocole DICOM standardisé.

E - Etape de planification du geste opératoire
1/ Le logiciel de navigation récupère cet examen et réalise automatiquement le recalage, étape préliminaire nécessaire à la phase de navigation.
2/ Le radiologue utilise le porte-aiguille non stérile pour a) naviguer dans les images scanner du patient, b) réaliser la planification de son geste et c) identifier le point d'entrée sur la peau du patient autour duquel le champ stérile nécessaire à la procédure interventionnelle sera réalisé.

F - Réalisation du champ stérile :
1/ Il est réalisé suivant le protocole habituel.
2/ De plus, afin de travailler dans des conditions optimales de localisation, le radiologue positionne le précédent référentiel hybride au niveau de la zone de travail, ce référentiel ayant été naturellement et préalablement placé dans une housse stérile.
3/ Un nouvelle TDM ciblée sur la zone de travail est alors réalisée. Cet examen est l'examen de référence pour la planification du geste sur la station de navigation. Suite à cette étape, le patient ainsi que le radiologue sont dans des conditions stériles.

G - Etape de navigation :
1/ Le radiologue utilise le porte aiguille stérile équipé du capteur magnétique inséré dans sa housse stérile.
2/ Le radiologue navigue dans les images scanner du patient dans des conditions stériles.

A tout moment, le radiologue peut décider de réaliser un examen scanner de contrôle afin de vérifier le bon positionnement de son instrument. Dans ce cas, la boucle décrite ci-dessous sera réalisée autant de que le radiologue le souhaite et jusqu'au bon positionnement final de son instrument:

H - Boucle :
1/ Réalisation d'un scanner de contrôle
2/ L'examen est poussé sur la station de navigation

3/ Le logiciel de navigation récupère l'examen et réalise automatiquement le recalage
4/ Le radiologue utilise le porte aiguille stérile équipé du capteur magnétique inséré dans sa housse stérile pour naviguer dans les images scanner.

Dans le cas d'une défaillance du dispositif, le radiologue pourra à tout moment abandonner la procédure naviguée et basculer vers la procédure classique, sans aucune conséquence concernant la qualité de son intervention et la sécurité du patient.

Lors de la réalisation de ce protocole opératoire, deux variantes sont envisagées au cours des étapes F pour le positionnement du référentiel hybride nécessaire à la réalisation du geste interventionnel en stérile.

- **Variante n°1 :** Au lieu de déplacer le premier référentiel hybride, le radiologue place un second référentiel (pour lequel les précautions de stérilité ont été préalablement prises) au niveau de la zone de travail.
- **Variante n°2 :** Le référentiel hybride de l'étape C2 est bien placé d'emblée pour la réalisation du geste interventionnel en stérile (pas de gêne à la réalisation du champ stérile, et suffisamment proche du point à ponctionner). Il peut être inutile de le déplacer et de le mettre dans la housse stérile. **Les étapes F2, F3 sont alors shuntées.** La TDM ciblée n'est pas effectuée. C'est l**a TDM initiale de repérage qui sert dans ce cas de référence pour la planification du geste sur la station.**

4.2. Objectif principal et critère de jugement principal

Objectif principal :
Comparer la précision de mise en place ***d'une aiguille d'anesthésie locale*** sous contrôle tomodensitométrique dans le cadre d'intervention de radiologie interventionnelle pour les deux pratiques suivantes : intervention avec la station de navigation chirurgicale (Groupe NAV – nouveau traitement) et intervention sans station de navigation chirurgicale (Groupe CT – traitement de référence).

Détails :
Il s'agit d'évaluer ***la précision de la mise en place de l'aiguille d'anesthésie locale :***
Elle est déterminée pour chacune des deux méthodes et ceci de façon indépendante. L'étape de repérage est commune à tous les gestes et se déroule toujours de façon globalement identique. Lors de cette phase, il sera demandé à l'opérateur de positionner l'aiguille d'anesthésie locale en supposant prendre le trajet définitif allant jusqu'à la cible. L'aiguille d'anesthésie locale n'atteignant pas la cible, on considérera que la trajectoire de cette aiguille correspond à la trajectoire définitive.

Deux cas de figure sont envisagés :

1. le patient n'a pas bougé entre la planification et la réalisation effective du geste : les repères initiaux et ceux au moment de la détermination de la trajectoire sont inchangés (cf. figure 1). La **distance maximale *d*** entre la trajectoire planifiée (bleue) et la trajectoire réalisée, définie par

l'aiguille d'anesthésie locale (rouge), est déterminée dans l'espace 3D à partir des données acquises.

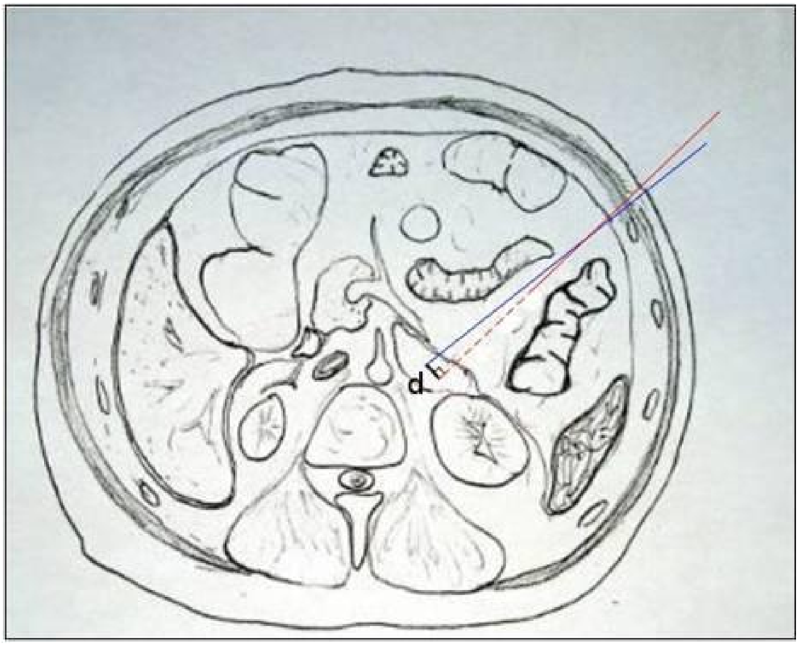

*Figure 1 : distance maximale **d** entre la trajectoire planifiée (bleu) et l'extrémité de l'aiguille d'anesthésie locale prolongée jusqu'à la cible (rouge)*

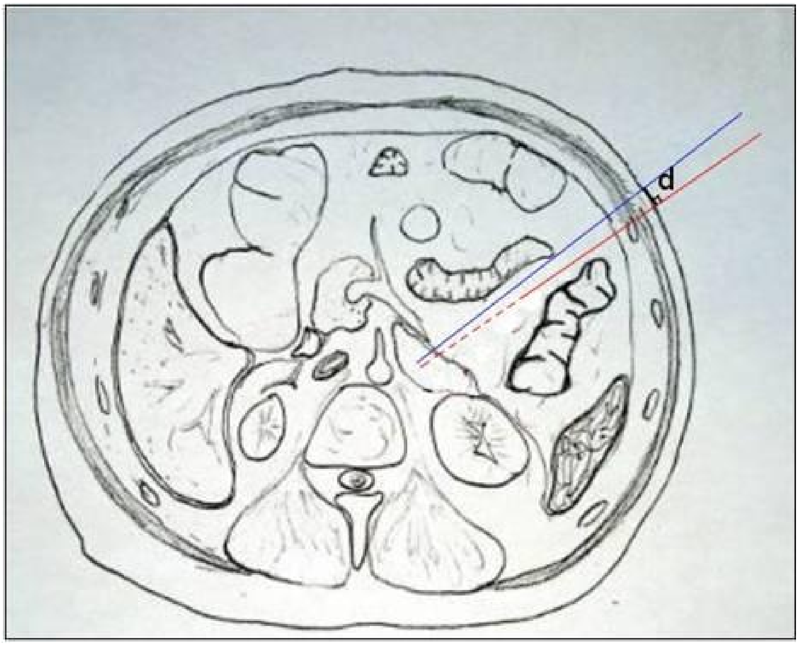

*Figure 2 : distance maximale **d** entre la trajectoire planifiée (bleu) et l'extrémité de l'aiguille d'anesthésie locale prolongée jusqu'à la cible (rouge)*

2. le patient a bougé entre la planification et la réalisation effective du geste : les repères sont modifiés, la cible n'a plus les mêmes coordonnées (figure 3). Il faut tenir compte de cette modification car une bonne trajectoire peut manquer la cible (trait rouge) et

inversement une mauvaise trajectoire (trait vert) peut l'atteindre, tout en traversant des organes à éviter (comme le colon dans cet exemple). Le bougé du patient sera estimé d'une part de manière subjective, et d'autre part de manière objective par comparaison de la position du référentiel hybride dans les images scanographiques qui ont servi à la planification avec les images scanographiques de contrôle. Remarque : cette double estimation permettra de confronter le ressenti du radiologue avec une réalité objective.

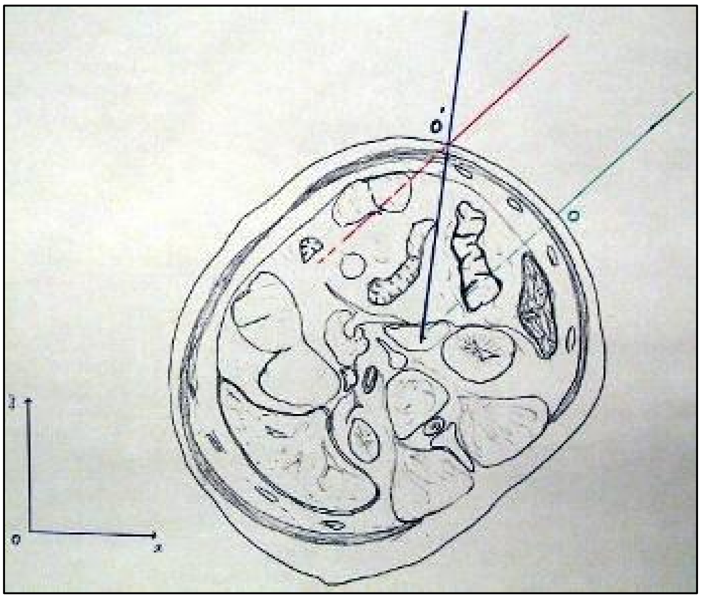

Figure 3 : Le patient a bougé. Les coordonnées de la cible, du point d'entrée et de la trajectoire sont modifiées dans le même repère.

Critère de jugement principal (étape 1) :

Distance maximale **d** entre la trajectoire planifiée et la trajectoire de ***l'aiguille d'anesthésie locale extrapolée***.

On disposera des données suivantes :

- Trajectoire planifiée : point d'entrée, point cible et équation de la droite (repère du scanner),
- Trajectoire réalisée : point d'entrée, point cible et équation de la droite (repère du scanner).

Ces mesures concernent uniquement l'étape de repérage.

Pour la méthode classique, on compare la trajectoire planifiée sur la console scanner avec la trajectoire de l'aiguille d'anesthésie locale effectivement obtenue sur l'acquisition scanner de contrôle.

Avec la station de navigation, on compare la trajectoire planifiée sur la station avec la trajectoire de l'aiguille d'anesthésie locale effectivement obtenue sur l'acquisition scanner de contrôle. On détermine ainsi la précision propre aux deux méthodes de façon indépendante.

4.3. Objectifs secondaires et critères de jugement secondaires

Objectif 1 : Comparer la précision de mise en place de l'aiguille de ponction

L'objectif principal de cette étude a concerné uniquement la phase de repérage (mise en place de l'aiguille d'anesthésie locale) puisque c'est la seule l'étape globalement identique entre les deux méthodes, et ceci,

indépendamment du patient ou du geste interventionnel réalisé. Cependant nous souhaitons aussi évaluer la précision de mise en place de l'aiguille de ponction. Après la phase de repérage, le geste interventionnel se poursuit avec la même technique utilisée pour la mise en place de l'aiguille d'anesthésie locale ce qui nous permettra de comparer la précision de mise de place de l'aiguille définitive de ponction entre le groupe CT et le groupe Nav.

Critère de jugement 1 (étape 2) :

- Distance maximale **d** entre la trajectoire planifiée et la trajectoire effectivement réalisée par l'aiguille de ponction.

On disposera des données suivantes :

- Trajectoire planifiée : point d'entrée, point cible et équation de la droite (repère du scanner),
- Trajectoire réalisée : point d'entrée, point cible et équation de la droite (repère du scanner),

Objectif 2 : comparaison des durées d'intervention

La comparaison des durées d'intervention entre les deux groupes NAV et CT prendra en compte de facteurs potentiels de confusion. Les données temporelles des différentes étapes du protocole opératoire seront recueillies :

- H0 : Première acquisition TDM,
- H1 : Aiguille d'anesthésie locale en place et acquisition de contrôle effectuée
- H2 : Aiguille de ponction en place et acquisition de contrôle effectuée (1ère acquisition de contrôle pour laquelle le radiologue pense avoir atteint sa cible)
- H3 : Dernier scanner de contrôle pour les éventuelles complications interventionnelles

Critère de jugement 2:

- Durée de la phase de repérage (étape 1) Δ1=H1-H0
- Durée du geste interventionnel (étape 2) Δ2=H2-H1
- Durée d'intervention Δ3=H3-H0

Objectif 3 : dose d'irradiation

Comparer la dose totale d'irradiation entre les deux groupes NAV et CT

Critère de jugement 3:

- Produit Dose Longueur en mGray*cm durant Δ1 (De H0 à H1 inclus)
- Produit Dose Longueur en mGray*cm durant Δ2 (De H1 exclu à H2 inclus)
- Produit Dose Longueur total en mGray*cm (De H0 à H3 inclus)
- Nombre de séries TDM effectués

Objectif 4 : Evaluation de la satisfaction de l'opérateur

Les critères satisfaction de l'opérateur comprennent :

1. La précision des gestes ressentie par l'opérateur

2. Les contraintes de réalisation : par exemple, maniabilité des outils, contraintes liées au dispositif de repérage comme la mise en place de l'objet de référence, mise en place du champ stérile, artéfacts générés par l'objet de référence
3. L'intérêt des deux techniques lorsque le plan de la trajectoire optimale n'est pas le plan transversal d'acquisition
4. La liberté de mouvements
5. La confiance ressentie par l'opérateur dans la réalisation de son geste
6. Score global de satisfaction concernant le déroulement et la réalisation de l'intervention

Chaque critère sera évalué à l'aide, soit d'une échelle visuelle analogique, soit de questions à choix unique.

Critère de jugement 4 :

- Score de satisfaction global (groupe NAV et groupe CT)

Objectif 5 : Echec ou Réussite du geste interventionnel réalisé

Comparer les résultats définitifs (échec ou réussite) des gestes effectués avec la station de navigation avec ceux effectués avec la méthode classique. Les échecs/réussites seront définis à partir de données objectives obtenues suite à la réalisation du geste et fonction de l'indication : drain en place sur contrôle radiologique, résultat anatomopathologique du tissu biopsié, etc…

Critère de jugement 5 :

- Echec ou Réussite du geste définitif réalisé

Objectif 6 : Nombre de tentatives

Les nombres de tentatives pour atteindre la cible, entre les groupes Nav et CT seront dénombrés. On comptabilise le nombre total de tentatives, aussi bien dans les phases de repérage avec positionnement de l'aiguille d'anesthésie locale que lors de la réalisation du geste final.

Critère de jugement 6 :

- Nombre de tentatives de ponction jusqu'à réussite du geste

Objectif 7 : Complication

Les complications survenues lors de la réalisation du geste seront comparées entre les deux groupes.

Nous proposons d'utiliser **la classification de la SIR (Society of Interventionnal Radiology)** utilisée en radiologie interventionnelle :

TABLE 29.1 SOCIETY OF INTERVENTIONAL RADIOLOGY (SIR) CLASSIFICATION SYSTEM FOR COMPLICATIONS BY OUTCOME

Minor Complications
A. No therapy, no consequence
B. Nominal therapy, no consequence; includes overnight admission for observation only
Major Complications
C. Require therapy, minor hospitalization(<48 hours)
D. Require major therapy, unplanned increase in level of care, prolonged hospitalization (>48 hours)
E. Permanent adverse sequelae
F. Death

Reprinted from Sacks D, McClenny TE, Cardella JF, Lewis CA. Society of Interventional Radiology clinical practice guidelines. *J Vasc Interv Radiol.* 2003;14:S199–S202, with permission from Elsevier.

Complications mineures :

A. Aucun traitement, aucune conséquence.
B. Mise en place d'un traitement léger (antalgique mineur par exemple), aucune conséquence ; surveillance médicale sans augmentation de la durée d'hospitalisation.

Complications majeures :

C. Mise en place d'un traitement lourd (transfusion par exemple), hospitalisation de courte durée (<48 heures).
D. Mise en place d'un traitement lourd, augmentation du niveau de soin (passage en réanimation, nécessité d'un geste chirurgical), hospitalisation (>48 heures).
E. Présences de séquelles irréversibles.
F. Décès.

Critère de jugement 7 :
- Nombre de complications immédiates et post-interventionnelles
- Type de complications immédiates et post-interventionnelles (grade A à F de la classification de la SIR)

Objectif 8 : Estimation du service médical attendu et rendu (SMA et SMR)

A partir des différents paramètres précédemment évalués, une première estimation du service médical attendu et potentiellement rendu sera réalisée.

Objectif 9 : Prise en compte de potentiels facteurs de confusion

Différents facteurs potentiels de confusion seront relevés au cours de cette recherche biomédicale :

- Expérience de l'opérateur (novice/expert)
- Nombre n d'interventions naviguées déjà réalisées par l'opérateur
- Evaluation subjective de l'accessibilité de la cible (aisé/difficile et/ou profonde/superficielle)
- Indication interventionnelle
- Position du patient (décubitus latéral gauche, décubitus latéral droite, ventral, dorsal)
- Les mouvements éventuels du patient (bougé/pas bougé)
- Modalité interventionnelle dans le groupe navigué (placement du (des) référentiel(s) hybride(s) pour la planification : 1 référentiel utilisé immobile, 1 référentiel utilisé déplacé, 2 référentiels utilisés)

5. CONCEPTION DE LA RECHERCHE

5.1. Type d'étude

- Essai clinique de dispositif médical innovant
- Etude monocentrique
- Etude de phase : Equivalent de phase II/III médicamenteuse
- Prospectif
- Contrôlée
- Ouverte
- Essai en groupe parallèles
- Randomisée

5.2. Organisation générale de l'étude et schéma

5.2.1. Schéma expérimental

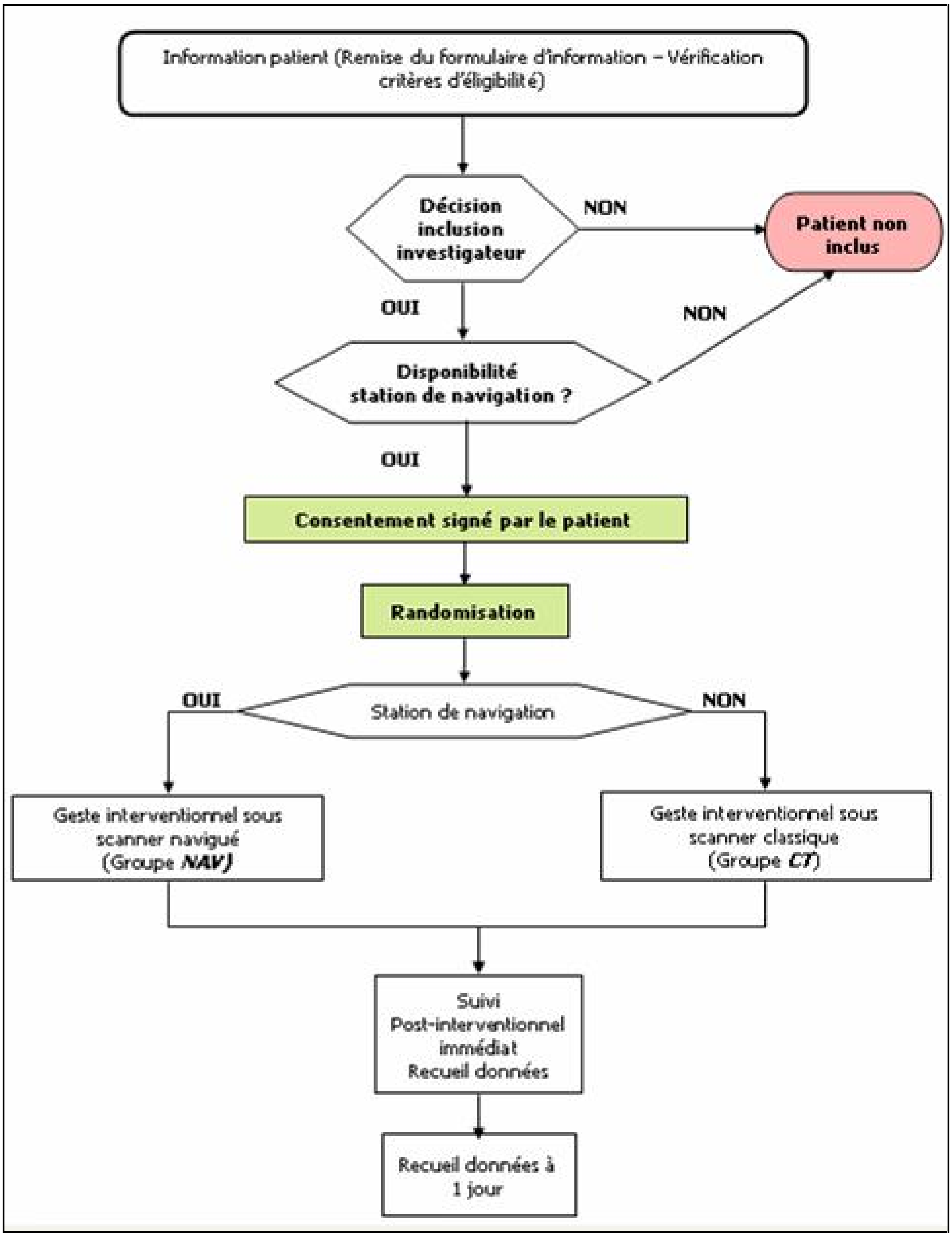

5.2.2. Déroulement pratique de l'étude

ETAPE 1 – Formation des radiologues interventionnels à l'utilisation de la station de navigation

Le dispositif médical à l'étude étant très intuitif, les opérateurs se formeront tout d'abord à son utilisation sur un fantôme (deux interventions minimum sur fantôme et en présence du formateur). Si l'opérateur en formation souhaite s'exercer davantage afin d'être plus à l'aise avec le système, il pourra naturellement le faire.

Suite à cette formation les inclusions pourront débuter. Afin de prévenir une éventuelle difficulté liée à la première utilisation du nouveau dispositif sur patient, les deux premières interventions seront réalisées en

présence du radiologue formateur. Selon le degré d'aisance ressenti par l'opérateur, les interventions suivantes pourront être réalisées en autonomie.

ETAPE 2 – Recrutement des patients dans le service de radiologie

Le recrutement des patients potentiels aura lieu lors de la pose de l'indication. Lors de cette décision, il sera remis au patient potentiellement incluable un formulaire d'information. Suite à un délai de réflexion, le patient, s'il souhaite participer à l'étude, signera le formulaire de consentement. Un exemplaire de ces documents sera remis au patient.

Avant toute inclusion, la disponibilité de la station de navigation sera naturellement vérifiée.

ETAPE 3 – Réalisation de l'intervention

Le patient, ayant ainsi accepté de participer à cette recherche biomédicale, sera alors pris en charge de manière « habituelle » dans le service de radiologie dans le cadre de ce protocole : randomisé dans l'un des deux groupes (CT, Nav), le geste interventionnel sera réalisé, soit de manière classique (groupe CT), soit guidé par la navigation (groupe Nav). Avec la méthode classique, l'introduction du matériel se fait uniquement sous contrôle scanner alors qu'avec la station de navigation, l'opérateur utilise les informations fournies par la station avec également la possibilité de contrôler l'avancée de l'aiguille dans le corps en réalisant des coupes scanner. Lors de la phase de repérage, l'opérateur a planifié l'introduction de l'aiguille et l'a introduite conformément à cette trajectoire. L'opérateur reprend ensuite cette trajectoire et poursuit son geste pour réaliser l'intervention souhaitée selon la modalité de initiale guidage (classique / naviguée).

Naturellement, à tout moment, s'il le souhaite, le patient pourra retirer son consentement et ne plus participer à cette recherche biomédicale.

ETAPE 4 – Suivi du patient en post-interventionnel immédiat

Lors du suivi post-interventionnel habituel du patient, le radiologue sera chargé de recueillir les éléments cliniques du patient inclus dans l'étude sur le cahier d'observation électronique.

Etape 5 – Evaluation clinique du patient lors de la visite de contrôle

Les patients qui bénéficient d'un geste interventionnel sont revus lors d'une visite de contrôle.

5.2.3. Procédures d'investigation menées et différences par rapport à la prise en charge habituelle

Cette étude n'entraîne aucune différence de prise en charge du patient.

5.3. Lieux de l'étude

CHU Grenoble : Clinique Universitaire de Radiologie et d'Imagerie Médicale (**CURIM**)

6. <u>CARACTERISTIQUES DES SUJETS</u>

6.1. Modalités de recrutement des sujets

Les patients, pour lesquels une indication du geste interventionnel a été retenue de manière consensuelle suite à une discussion entre radiologues, cliniciens et chirurgiens, seront recrutés dans le centre d'étude précédemment identifié.

6.2. Critères d'inclusion

Seront proposés pour l'étude les sujets répondant à chacun des critères suivants :

- Personne majeure,
- Personne affiliée à la sécurité sociale ou bénéficiaire d'un tel régime,
- Personne pour lesquelles une indication de geste interventionnel percutané diagnostique ou thérapeutique sous contrôle tomodensitométrique est posée. Ce geste sera effectué après un examen clinique dont les conclusions seront communiquées directement au patient.

6.3. Critères de non-inclusion

Ne pourront pas être inclus les sujets répondant à au moins un des critères suivants :

- Femmes enceintes, parturientes, et mères qui allaitent,
- Personnes privées de liberté par décision judiciaire ou administrative,
- Personnes hospitalisées sans consentement (HO ou HDT),
- Personnes majeures faisant l'objet de protection légale ou hors d'état d'exprimer leur consentement,
- Contre-indication habituelle à la radiologie interventionnelle
- Contre-indications habituelles à l'IRM. En particulier, l'utilisation du DM sera exclue pour les patients porteurs d'un matériel non compatible IRM tel qu'un stimulateur cardiaque.
- Seront aussi exclus les patients qui présentent des corps étrangers ferromagnétiques intracorporels proches de la zone de travail du radiologue et susceptibles d'interagir avec le DM, même si ces corps étrangers ne constituent pas une contre-indication à l'IRM.

6.4. Traitements associés autorisés

L'indication interventionnelle ayant été posée de manière consensuelle, tous les traitements qui ne contre-indiquent pas l'acte interventionnel sont autorisés.

7. VARIABLES MESURÉES ET MÉTHODES DE MESURE

7.1. Paramètres cliniques

Les principaux paramètres cliniques recueillis sont les suivants :

- Sexe,
- Age

7.2. Paramètres para-cliniques

Les paramètres recueillis seront relatifs respectivement à :

- Paramètres d'hémostases : TP, TCA, INR, Plaquettes
- l'indication interventionnelle,
- l'accessibilité de la cible,
- la position du patient lors de l'intervention,

- l'expérience de l'opérateur (novice/expert)
- le nombre n d'interventions naviguées déjà réalisées par l'opérateur
- la précision du geste en phase de repérage (comparaison des trajectoires planifiées et réalisées pour l'aiguille d'anesthésie locale)
- la précision du geste au cours du geste interventionnel (comparaison des trajectoires planifiées et réalisées pour l'aiguille de ponction)
- les durées interventionnelles (phase de repérage et totale)
- la dose d'irradiation (Produit dose longueur)
- la réussite de l'intervention (résultats d'anatomopathologie)
- le nombre de tentatives de ponction jusqu'à la réussite du geste
- les complications interventionnelles
- l'évaluation qualitative et comparative de la nouvelle technique (questionnaire d'évaluation)
- les limitations rencontrées avec l'utilisation de la station de navigation (questionnaire d'évaluation)
- bougé du patient
- modalité interventionnelle dans le groupe navigué (référentiel hybride)

7.3. Données recueillies directement dans le cahier d'observation

En dehors de l'examen clinique prévu dans le cadre de l'étude, l'ensemble des données décrites ci-dessus sera inscrit directement dans le cahier d'observation électronique de l'étude.

8. TRAITEMENT OU PROCEDURES COMPAREES

8.1. Traitement testé

Le « traitement » testé est l'utilisation de la station de navigation au cours d'un geste interventionnel, diagnostique ou thérapeutique, sous scanner.

8.2. Traitement de référence

Le « traitement de référence » est l'intervention classique pour geste interventionnel, diagnostique ou thérapeutique, sous scanner.

8.3. Méthode d'allocation aléatoire (randomisation centralisée)

Après vérification des critères d'inclusion et de non-inclusion, la randomisation centralisée sera réalisée électroniquement par une société de service externe aux CHU - en particulier, un site de randomisation sera mis à disposition des investigateurs 24h/24, ceci afin d'avoir un outil adapté au mode de vie des radiologues.

9. RECUEIL ET GESTION DES DONNEES

9.1. Recueil des données

Toutes les données de l'interrogatoire et des examens cliniques devront être présentées dans un dossier spécifique au protocole, consultable par le promoteur, qui constituera les données sources.

Les cahiers d'observation seront remplis et signés par l'investigateur.

Par son accord de participation, l'investigateur s'engage à conduire la recherche dans le strict respect du protocole expérimental, des "Bonnes Pratiques Cliniques" et des dispositions législatives et réglementaires en vigueur. Il se porte garant de l'authenticité des données recueillies dans le cadre de l'étude et accepte les dispositions légales autorisant le promoteur de l'étude à mettre en place un contrôle de la qualité.

L'analyse statistique ne sera effectuée qu'après vérification de la saisie et de la cohérence des données. Les données seront archivées par le coordonnateur.

9.2. Monitorage

Un monitorage des données sera effectué sur toutes les données par un Attaché de Recherche Clinique ou l'ingénieur du CIC-IT du CHU de Grenoble. Il portera sur 10% des cahiers d'observation tirés au sort dans chaque centre recruteur.

L'ensemble des consentements sera vérifié, ainsi que les critères d'inclusion/non inclusion, et les évènements indésirables.

Un compte rendu de monitorage sera rédigé par l'ARC et/ou l'ingénieur et conservé dans le dossier d'étude.

10. ANALYSE STATISTIQUE DES PARAMÈTRES MESURÉS

10.1. Comité de pilotage

Un comité de pilotage sera constitué dès le début de cette étude. Il comprendra le Dr Ivan Bricault, le Dr Alexandre Moreau-Gaudry et le Pr Jean-Luc Bosson. Ce comité aura pour fonction de se prononcer, en particulier à partir des résultats obtenus suite à l'analyse intermédiaire, sur la poursuite ou l'arrêt de l'étude. Il sera aussi en charge de la gestion des modifications apportées au plan statistique initial.

10.2. Stratégie d'analyse des données

10.2.1. Description de la population étudiée

Les paramètres quantitatifs seront décrits par leurs moyennes ± écart-type, médiane [percentile à 25% - percentile à 75%], maximum et minimum. Les paramètres qualitatifs seront exprimés en effectif et pourcentage assortis de leur intervalle de confiance à 95%.
La comparaison des 2 groupes d'étude (NAV et CT) sera réalisée afin de contrôler la bonne mise en œuvre de la randomisation et de décrire les éventuels facteurs de confusion.

10.2.2. Choix des risque ~ de première espèce et de la puissance

L'analyse des critères de jugement sera globale (CT vs NAV). De manière usuelle, le risque d'erreur α de première espèce est fixé à 5%, la puissance à 90%.
Une analyse intermédiaire est prévue un an après la date du premier inclus afin de s'assurer du bon déroulement de l'étude (avec en particulier, une première évaluation de la station de navigation et de sa compatibilité avec ses spécifications ainsi qu'une évaluation du recrutement effectif par rapport au recrutement prévisionnel). Afin de conserver un seuil global à 5% lors de l'analyse terminale, un seuil à 0,1% sera retenu lors de la réalisation de l'analyse intermédiaire [19].

10.2.3. Description des critères de jugement étudiés

Objectif principal

Conformément aux données obtenues lors des études précliniques (cf. Annexe A), la moyenne des distances de l'extrémité de l'aiguille à la cible dans le groupe Nav est, de manière significative, différente de celle du groupe CT. Cette recherche biomédicale a donc été construite comme un essai de comparaison bilatéral de moyenne des groupes Nav versus CT relativement au critère distance.

L'analyse sera réalisée en intention de traitée. Elle pourra être complétée d'une analyse en per-protocole.

Compte tenu de l'absence de double aveugle, des méthodes d'ajustement *a posteriori* (modélisation linéaire multiple) permettront d'ajuster la variable à expliquer sur les éventuels facteurs de confusion précédemment identifiés.

Objectif secondaire

Chaque critère de jugement fera l'objet d'une analyse univariée permettant de comparer les 2 groupes étudiés. Le test t de Student sera mis en œuvre pour comparer les variables quantitatives. Dans le cas où les conditions de ce test ne seraient pas vérifiées, des tests non paramétriques seront utilisés. Les liaisons potentielles entre paramètres qualitatifs seront objectivées à l'aide du test du chi-deux ou test exact de Fisher si les effectifs sont insuffisants. Puis, compte tenu de l'absence de double aveugle, des méthodes d'ajustement *a posteriori* (modélisation linéaire multiple) pourront être mises en œuvre pour ajuster les résultats sur d'éventuels facteurs de confusion.

Les courbes d'apprentissage des différents intervenants seront représentées graphiquement.

10.3. Calcul du nombre de sujets

Le calcul du nombre de sujet est basé sur la validation de l'objectif principal à partir des résultats des données précliniques. Déterminé de manière usuelle, les hypothèses suivantes ont été utilisées (cf. Annexe A) :

- $\mu_{CT} = 13$
- $\mu_{Nav} = 6$
- $\sigma = Max(11,3 ; 4) = 11,3$
- $\alpha = 0,05$
- puissance = 0,9

soit un nombre de sujet nécessaire de 56 par groupe. Le nombre total théorique de patient à inclure est donc de 112. En considérant 5% de perdu de vue potentiel, le nombre de sujets effectivement à inclure est de 59 par groupe, soit approximativement 120 au total.

10.4. Responsable des analyses

Le responsable des analyses sera l'une des personnes suivante du CHU de Grenoble :

- Docteur Ivan Bricault, Maître de Conférences, Praticien Hospitalier du CURIM,

- Docteur Alexandre Moreau-Gaudry, Assistant Hospitalo Universitaire au CIC-IT du Département de Méthodologie et d'Information en Santé (DMIS),
- Monsieur Jean-Louis Quesada, biostatisticien du CIC du CHU de Grenoble.

10.5. Lieu d'analyse des données et logiciels utilisés

Le CURIM ou le CIC-IT de Grenoble seront les lieux d'analyse des données anonymisées. Cette analyse aura lieu conformément aux bonnes pratiques d'analyse statistique après gel de la base de données et sera réalisée avec le logiciel GNU R..

RÉFÉRENCES BIBLIOGRAPHIQUES

1. General Principles for Evaluation of New Interventional Technologies and Devices. Sacks D, Marinelli D, Martin L et al. J Vasc Interv Radiol 2003 ;14 :S391-S394

2. Quality Improvement Guidelines for Image-guided Percutaneous Biopsy in Adults. Cardella JF, Bakal CW, Bertino RE et al. J Vasc Interv Radiol 2003 ;14 :S227-S230

3. Quality Improvement Guidelines for Adult Percutaneous Abscess and Fluid Drainage

Bakal CW, Sacks CD, Burke DR et al. J Vasc Interv Radiol 2003 ;14 :S223-S225

4. Society of Interventional Radiology Quality Improvement Guidelines for Percutaneous Vertebroplasty. McGraw JK, Cardella J, Barr JD et al. J Vasc Interv Radiol 2003 ;14 :S311-S315

5. CT-guided procedures: evaluation of a phantom system to teach accurate needle placement. Dimmick S, Jones M, Challen J Clin Radiol. 2007 Feb;62(2):166-71.

6. CT-guided percutaneous transthoracic biopsy in the evaluation of undetermined pulmonary lesions. Lourenco R, Camacho R, Barata MJ Rev Port Pneumol. 2006 Sep-Oct;12(5):503-24

7. CT-guided bone biopsy: Initial experience with a commercially available hand held Black and Decker drill. Buckley O, Benfayed W, Geoghegan T. Eur J Radiol. 2007 Jan;61(1):176-80.

8. Augmented reality visualization for CT-guided interventions: system description, feasibility, and initial evaluation in an abdominal phantom. Das M, Sauer F, Schoepf UJ, Radiology. 2006 Jul;240(1):230-5.

9. Accuracy and complications in computed tomography fluoroscopy-guided needle biopsies of lung masses. Heck SL, Blom P, Berstad A. Eur Radiol. 2006 Jun;16(6):1387-92.

10. Out-of-plane computed-tomography-guided biopsy using a magnetic-field-based navigation system. Wallace MJ, Gupta S, Hicks ME. Cardiovasc Intervent Radiol. 2006 Jan-Feb;29(1):108-13

11. Computed-tomography-guided punctures using a new guidance device. Magnusson A, Radecka E, Lonnemark M et al. Acta Radiol. 2005 Aug;46(5):505-9.

12. Complications of fluoroscopy-, ultrasound-, and CT-guided percutaneous interventional procedures. Dupas B, Frampas E, Leaute F, J Radiol. 2005 May;86:586-98.

13. Design and analysis of randomized clinical trials requiring prolonged observation of each patient. I. Introduction and design. Peto R, Pike MC, Armitage P et al. Br J Cancer. 1976 Dec;34(6):585-612.

14. Electromagnetic tracking for abdominal interventions in computer aided surgery. Zhang H, Banovac F, Lin R et al. Comput Aided Surg. 2006 May;11(3):127-36.

15. Intérêt de l'utilisation d'une station de navigation dans le guidage des gestes effectués sous tomodensitométrie. Bing F, Bricault I, Sengel C, Ferretti G. In: Proceedings of the Third International Conference Surgetica'2007, Chambéry. Merloz P, Troccaz J, editors ; Sauramps Médical, 2007.

16. Needle biopsy of anatomically unfavorable liver lesions with an electromagnetic navigation assist device in a computed tomography environment. Banovac F, Wilson E, Zhang Het al. J Vasc Interv Radiol. 2006 Oct;17(10):1671-5.

17. Value of a laser guidance system for CT interventions: a phantom study. Jacobi V, Thalhammer A, Kirchner J. Eur Radiol. 1999;9(1):137-40.

18. A Navigation System for Minimally Invasive CT-Guided Interventions. Nagel M, Schmidt G, Petzold Ret al. Gerig Editors; MICCAI 2005, LNCS 3750, Springer-Verlag, pp. 33–40, 2005.

19. Peto R, Pike MC, Armitage P, Breslow NE, Cox DR, Howard SV, Mantel N, McPherson K, Peto J, Smith PG. Design and analysis of randomized clinical trials requiring prolonged observation of each patient. I. Introduction and design. Br J Cancer. 1976 Dec;34(6):585-612.

ANNEXE A : Résultats Précliniques – Critère principal

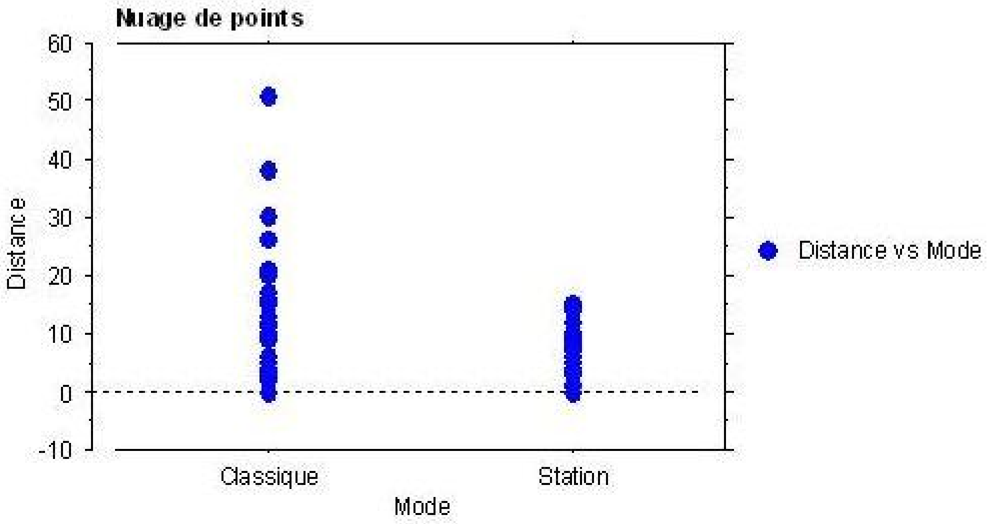

Statistiques descriptives

	Distance
Moy.	9,444
Dév. Std	9,125
Erreur Std	1,075
Nombre	72
Minimum	0,000
Maximum	51,000
# Manquants	0

Test-t séries non appariées pour Distance
Variable "groupe" : Mode
Ecart théorique = 0

	Ecart moyen	DDL	t	p
Classique, Station	7,056	70	3,536	,0007

Info. du groupe pour Distance
Variable "groupe" : Mode

	nombre	Moy.	Variance	Dév Std	Erreur Std
Classique	36	12,972	127,513	11,292	1,882
Station	36	5,917	15,793	3,974	,662
